# Phytoplankton and Microzooplankton Community Structure and Assembly Mechanisms in Northwestern Pacific Ocean Estuaries with Environmental Heterogeneity and Geographic Segregation

**DOI:** 10.1128/spectrum.04926-22

**Published:** 2023-03-20

**Authors:** Yi Sun, Hongjun Li, Xiaocheng Wang, Yuan Jin, Satoshi Nagai, Senjie Lin

**Affiliations:** a State Environmental Protection Key Laboratory of Coastal Ecosystem, National Marine Environmental Monitoring Center, Dalian, China; b Coastal and Inland Fisheries Ecosystems Division, Fisheries Technology Institute, Japan Fisheries Research and Education Agency, Kanagawa, Japan; c Department of Marine Sciences, University of Connecticut, Groton, Connecticut, USA; University of Thessalyd

**Keywords:** community assembly, dispersal limitation, environmental filtering, estuary ecosystems, phytoplankton, microzooplankton, zooplankton

## Abstract

Phytoplankton and microzooplankton are crucial players in marine ecosystems and first responders to environmental changes, but their community structures and how they are shaped by environmental conditions have rarely been studied simultaneously. In this study, we conducted an eDNA metabarcoding sequencing combined with multiple statistical methods to simultaneously analyze the phytoplankton and microzooplankton in Liaohe (LH) and Yalujiang (YLJ) estuaries. The major objective was to examine how plankton community structure and assembly mechanism may differ between two estuaries with similar latitudinal position and climate but geographical segregation and differential level of urbanization (more in LH). Clear differences in diversity and composition of phytoplankton and microzooplankton communities between LH and YLJ estuaries were observed. Richness of phytoplankton was significantly higher in LH than YLJ, while richness of microzooplankton was higher in YLJ. The magnitude of intrahabitat variations in phytoplankton communities was significantly stronger than that of microzooplankton. Some phytoplankton and microzooplankton taxa also showed interhabitat differences in their relative abundances. Phytoplankton showed a stronger geographic distance-decay of similarity than microzooplankton, while significant environmental distance-decay of similarity in microzooplankton was found in the less urbanized YLJ estuary. Community assembly of phytoplankton was, based on the neutral community models, driven primarily by stochastic processes, while deterministic processes contributed more for microzooplankton. Furthermore, we detected wider habitat niche breadths and stronger dispersal abilities in phytoplankton than in microzooplankton. These results suggest that passive dispersal shapes the phytoplankton community whereas environmental selection shapes the microzooplankton community.

**IMPORTANCE** Understanding the underlying mechanisms shaping a metacommunity is useful to management for improving the ecosystem function. The research presented in the manuscript mainly tried to address the effects of habitat geography and environmental conditions on the phytoplankton and microzooplankton communities, and the underlying mechanisms of community assembly in temperate estuaries. In order to achieve this purpose, we developed a metabarcoding sequencing method based on 18S rRNA gene. The phytoplankton and microzooplankton communities from two estuaries with similar latitude and climatic conditions but obvious geographical segregation and significant environmental heterogeneity were investigated. The results of our study could lay a solid foundation for ascertaining phytoplankton and microzooplankton communities in estuaries with obvious environmental heterogeneity and geographic segregation and mechanisms underlying community assembly.

## INTRODUCTION

Estuaries are the buffer areas of freshwater and seawater ecosystems, resulting in a unique environmental condition (1). The unique salt- and fresh-water exchange characteristic of estuaries fosters an extremely complex community of organisms with a high level of biodiversity (2). Estuaries have been shown to support a variety of essential ecological functions as well as provide services to humans (3). In recent years, estuarine ecosystems have increasingly demonstrated habitat degradation and biodiversity decline due to intensive anthropogenic activities and global climate change (4–6). From the metacommunity perspective, distance-decay of similarity is the most common biogeographic patterns that suggest potential driving mechanisms underlying the metacommunity assembly (7). Environmental selection and passive dispersal are two major ecological processes that determine the distance-decay patterns of communities in aquatic ecosystems (8). Environmental selection acts in such a way that community compositions are more similar between two locations with comparable environmental conditions (9). In contrast, passive dispersal leads to more similar community compositions between nearby locations than between those farther apart (10). Understanding the underlying mechanisms shaping a metacommunity is useful to management for improving the ecosystem function, but it is not fully clear in estuarine ecosystems.

Phytoplankton and microzooplankton occupy an important position in estuarine ecosystems, which are the basis of aquatic food web and significantly affect the ecosystem functions and services (11). Phytoplankton generate oxygen and fixes carbon dioxide through photosynthesis, making them critical for net primary production and biological carbon cycling (12). Meanwhile, phytoplankton as the food of herbivores directly or indirectly provide energy to organisms at higher trophic levels (13). Besides, many phytoplankton species can form harmful algal blooms, which cause damage to ecosystem functions and services (14). Microzooplankton mostly feed on phytoplankton and are responsible for transferring energy to higher trophic levels in the food chain (15). They are both first responders to environmental changes. Phytoplankton and microzooplankton can quickly respond to variations in environmental conditions, but they may get disparate consequences (16, 17). Both phytoplankton and microzooplankton are able to migrate into preferable habitats, among which phytoplankton only passively drift with the currents but microzooplankton have active mobility (18, 19). Therefore, it is important to consider the differences in community assembly mechanisms between phytoplankton and microzooplankton for a more comprehensive understanding of dynamic changes in biodiversity and ecosystem functions of estuaries.

**FIG 1:**
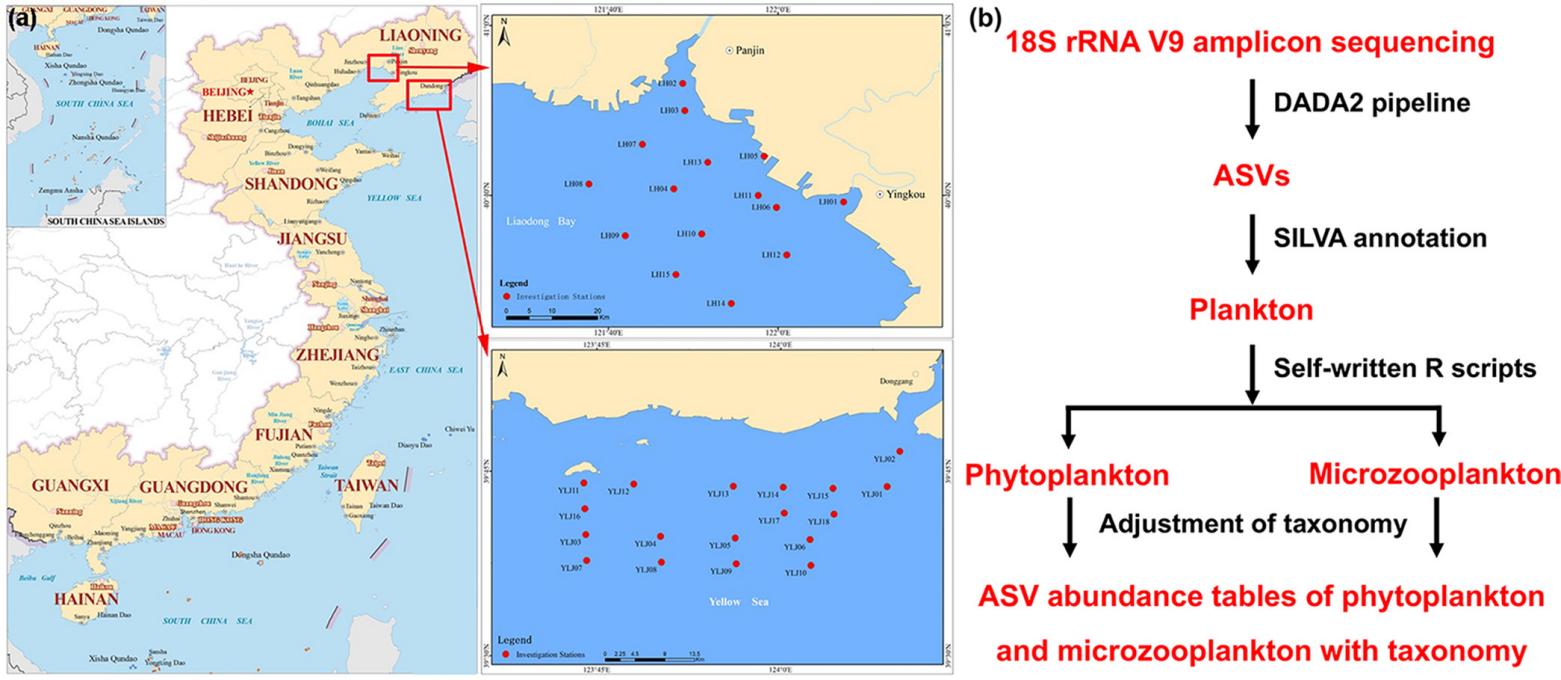
(a) Map of sampling sites. A total of 15 and 18 stations were established in the Liaohe (LH) and Yalujiang (YLJ) estuaries, respectively. (b) Sequencing and species annotation procedure.

Liaohe (LH) River and Yalujiang (YLJ) River are the two most important rivers flowing into the sea in the northeast of China. The estuaries of LH and YLJ are located on different sides of the Liaodong Peninsula (Fig. 1a) with similar latitude and climatic conditions. The LH River runs into the Liaodong Bay, a part of the Bohai Sea, which runs through a highly developed oil industrial city and tourist city. Coupling with large-scale offshore oil exploration activities, the LH estuary area has been reported to be the most contaminated area in China (20). In contrast, the YLJ River is at the border between China and North Korea, which runs into the North Yellow Sea. The YLJ estuary is an important shipping hub and large-scale offshore open mariculture area, which has potential eutrophication risks and heavy metal pollution (21). Both estuaries have been used as models in investigations to understand impacts of environmental variations on the local biodiversity (22). Our previous study has demonstrated the different regional species pools and environmental variables between LH and YLJ estuaries, corresponding to remarkably distinct bacterial and protist communities (23).

Studies on phytoplankton and microzooplankton in aquatic ecosystems can be performed with microscopy (17, 22, 24), but this traditional method is laborious and demands a high degree of taxonomic expertise (25). With the rapid development of next-generation sequencing, metabarcoding of environmental DNA (eDNA) has become a standard practice in the field of microbial ecology (26). Currently, increasing studies have applied eDNA metabarcoding to investigate plankton communities; however, most of them were focused on only phytoplankton or microzooplankton, not both (27, 28). In this study, we analyzed phytoplankton and microzooplankton communities simultaneously by metabarcoding sequencing of 18S rRNA gene in the LH and YLJ estuaries areas. Variations in assembly mechanisms of phytoplankton and microzooplankton communities between different studied areas were also evaluated. We hypothesized that the habitat heterogeneity and geographic segregation of LH and YLJ estuaries could induce distinct community assembly mechanisms and result in different plankton communities.

## RESULTS

### Annotation of phytoplankton and microzooplankton.

According to the sequencing and data processing pipeline (Fig. 1b), a total of 3,881,365 sequencing reads were obtained for 33 water samples in this study (Table S1), which clustered into 635 high-quality ASVs. Among them, 76 and 149 ASVs were annotated as phytoplankton and microzooplankton, respectively, and these accounted for 5,897 to 33,809 reads for phytoplankton and 1,908 to 44,845 reads for microzooplankton (Table S1). All phytoplankton and microzooplankton ASVs were successfully annotated at the phylum level (Fig. S1). A total of 6 and 20 phytoplankton phyla and microzooplankton lineages were identified, ranging from 2 to 5 and 1 to 6 for each sample, respectively (Table S2 and S3). Pyrrophyta, Bacillariophyta, and Charophyta accounted for most of the phytoplankton ASVs (Fig. S2a), whereas Ciliophora was the dominant microzooplankton lineage (Fig. S2b). About 75% and 50% ASVs were assigned phytoplankton and microzooplankton genera, respectively, but only 17.1% phytoplankton and 25.5% microzooplankton ASVs can be assigned to a species (Fig. S1). In addition, rarefaction curves based on the detected species numbers were all close to the plateau (Fig. S3), demonstrating that the amounts of effective sequencing reads were sufficient to represent the whole phytoplankton and microzooplankton communities. Moreover, the species accumulation curves were close to a straight line (Fig. S4), indicating that the number of samples collected in this study can fully represent the phytoplankton and microzooplankton communities in LH and YLJ estuaries.

### Differences in diversity and compositions of phytoplankton and microzooplankton communities between LH and YLJ estuaries.

**FIG 2:**
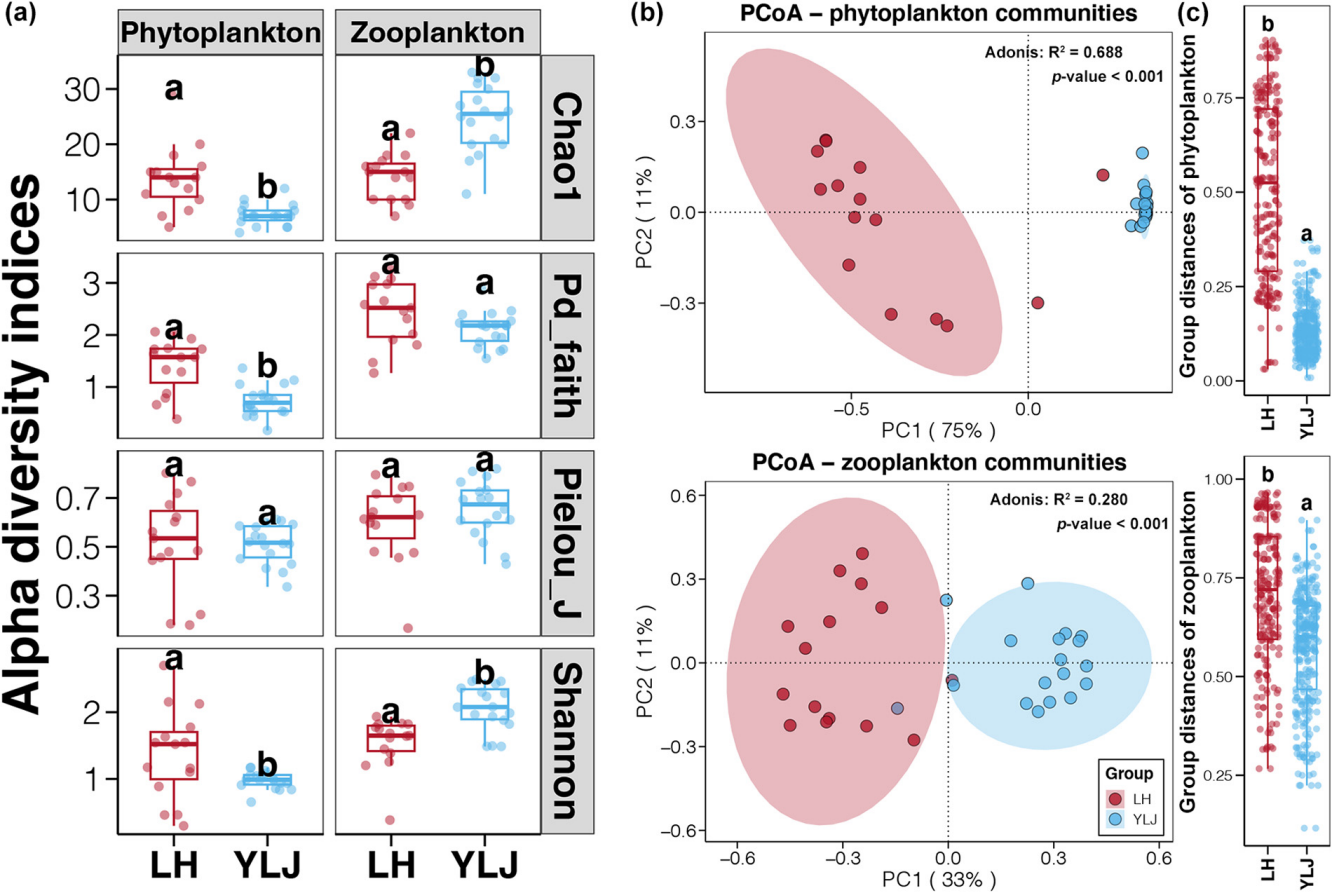
Differences in the alpha diversity indices of phytoplankton and microzooplankton communities between the LH and YLJ estuaries. (a) Alpha diversity indices of phytoplankton and microzooplankton communities in the LH and YLJ estuaries. (b) PCoA and Adonis tests of phytoplankton and microzooplankton communities between the LH and YLJ estuaries based on the Bray-Curtis distance, respectively. (c) Differences in Bray-Curtis distances of phytoplankton and microzooplankton communities between the LH and YLJ estuaries, respectively. Different lowercase letters above each box in the same subfigure represent significant differences between groups (*t* test, *P* < 0.05).

Phytoplankton communities in the LH estuary had significantly higher Chao1, Pd_faith, and Shannon indices than those in the YLJ estuary (*t* test, *P* < 0.05, Fig. 2a). In contrast, the Chao1 and Shannon indices of microzooplankton communities in YLJ were significantly higher than those in LH (*t* test, *P* < 0.05, Fig. 2a). No significant difference was found in Pielou_J indices between LH and YLJ for either phytoplankton or microzooplankton communities (*t* test, *P* > 0.05, Fig. 2a). These results indicated that there was no difference in community evenness between LH and YLJ, but the richness of phytoplankton and microzooplankton was higher in LH and YLJ, respectively.

Bray-Curtis distance was used to evaluate the variations of phytoplankton and microzooplankton communities between the LH and YLJ estuaries. Communities from different habitats were separately clustered and distributed on different sides of PC1, which explained 75% and 33% of the total variations in phytoplankton and microzooplankton, respectively (Fig. 2b). Significant between-region differences in composition of both phytoplankton and microzooplankton communities were also confirmed (Adonis test, *P* < 0.05). The Bray-Curtis distances of phytoplankton and microzooplankton communities in LH were significantly larger than those in YLJ (*t* test, *P* > 0.05, Fig. 2c). Moreover, intrahabitat distances of phytoplankton and microzooplankton communities in LH were similar, while in YLJ, the distances of microzooplankton communities were more pronounced relative to distances of phytoplankton communities (Fig. 2b and c). These findings indicated the differences in phytoplankton and microzooplankton compositions between the LH and YLJ estuaries and stronger variations in phytoplankton.

### Geographic differences in phytoplankton and microzooplankton.

**FIG 3:**
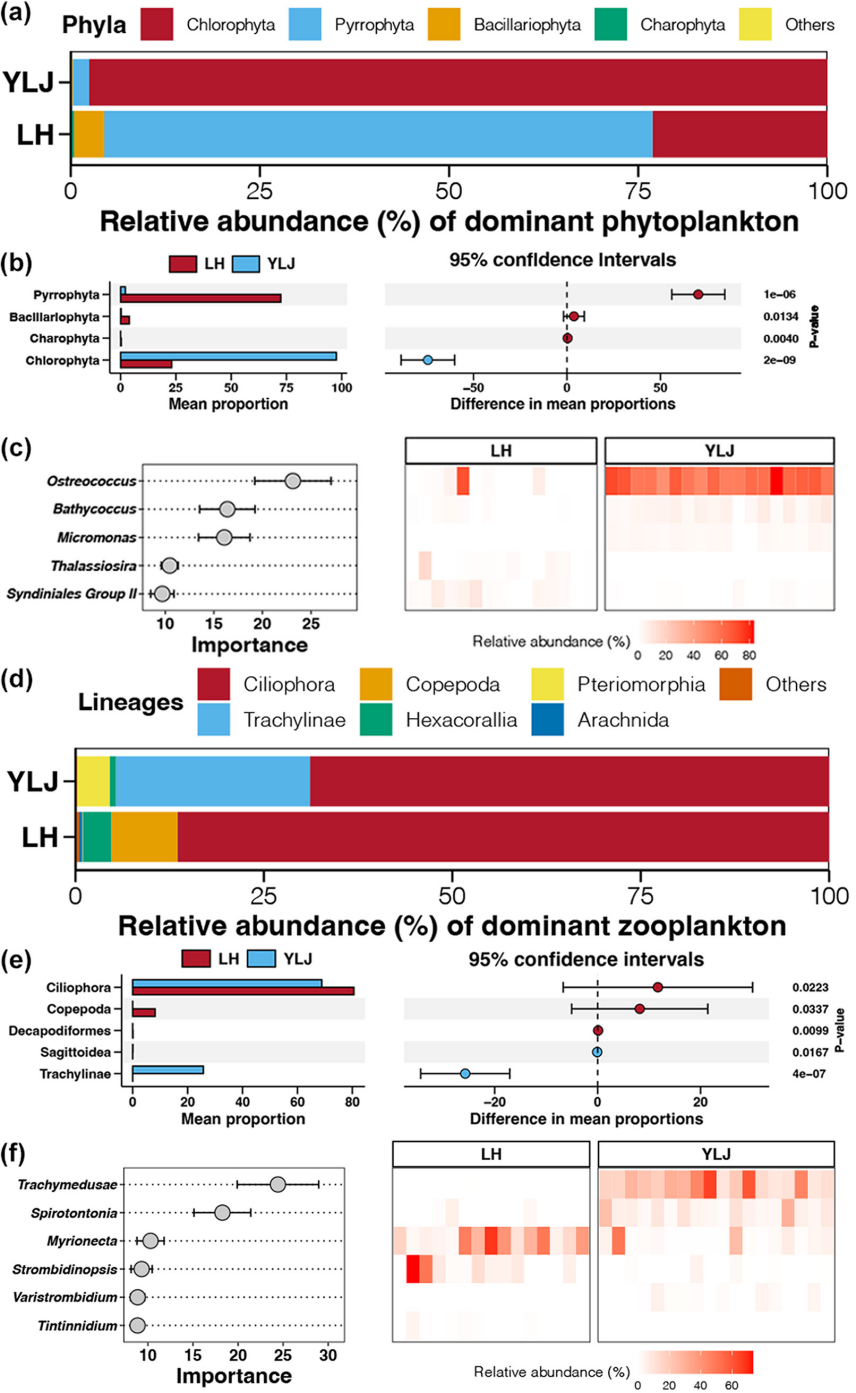
Characteristics of dominant phytoplankton and microzooplankton taxa in the LH and YLJ estuaries. (a) Relative abundance of dominant phytoplankton phyla in LH and YLJ. (b) Phytoplankton phyla with significantly different abundances between LH and YLJ. (c) Importance of key phytoplankton genera and their abundance distribution of key phytoplankton genera identified by the random forest method for distinguishing the LH and YLJ samples. (d) Relative abundance of dominant microzooplankton lineages in LH and YLJ. (e) Microzooplankton lineages with significantly different abundances between LH and YLJ. (f) Importance of key microzooplankton genera and their abundance distribution of key microzooplankton genera identified by the random forest method for distinguishing the LH and YLJ samples.

For phytoplankton, Pyrrophyta and Chlorophyta were dominant phyla in the LH and YLJ estuaries, respectively, followed by Bacillariophyta (Fig. 3a). The relative abundances of Pyrrophyta, Bacillariophyta, and Charophyta were significantly higher in LH than YLJ, whereas Chlorophyta was significantly more abundant in YLJ (Wilcox Rank-Sum test, *P* < 0.05, Fig. 3b). At the genus level, *Paragymnodinium* and *Ostreococcus* were the most dominant genera in the LH and YLJ estuaries, respectively (Fig. S5a). A random forest model was further constructed to recognize key taxa for predicting the origins of phytoplankton communities based on the genus-level data sets. Five phytoplankton genera, including *Ostreococcus*, *Bathycoccus*, *Micromonas*, *Thalassiosira*, and *Syndiniales* Group II, were identified to distinguish the phytoplankton communities between the LH and YLJ estuaries (Fig. 3c). The random forest model based on phytoplankton genera showed 93.94% accuracy for discrimination LH and YLJ samples (Fig. S6a).

For microzooplankton, Ciliophora was the dominant lineage in both the LH and YLJ estuaries, followed by Copepoda, Tetrapoda, and Hexacorallia in LH, and Trachylinae and Pteriomorphia in YLJ (Fig. 3d). Trachylinae and Sagittoidea were significantly more abundant in LH estuaries (Wilcox Rank-Sum test, *P* < 0.05, Fig. 3e). In contrast, significantly higher abundances of Ciliophora, Copepoda, and Descapodiformes were found in YLJ (Wilcox Rank-Sum test, *P* < 0.05, Fig. 3e). At the genus level, *Mesodinium* and *Trachymedusae* were the most dominant microzooplankton genera in the LH and YLJ estuaries, respectively (Fig. S5b). Based on the Random Forest model, six microzooplankton genera were chosen, including *Trachymedusae*, *Spirotontonia*, *Myrionecta*, *Strombidinopsis*, *Variatrombidium*, and *Tintinnidium* to distinguish the microzooplankton communities between the LH and YLJ estuaries (Fig. 3f). The model recorded an accuracy of 100% for the microzooplankton community differentiation (Fig. S6b).

### Distance-decay of similarity for phytoplankton and microzooplankton communities.

**FIG 4:**
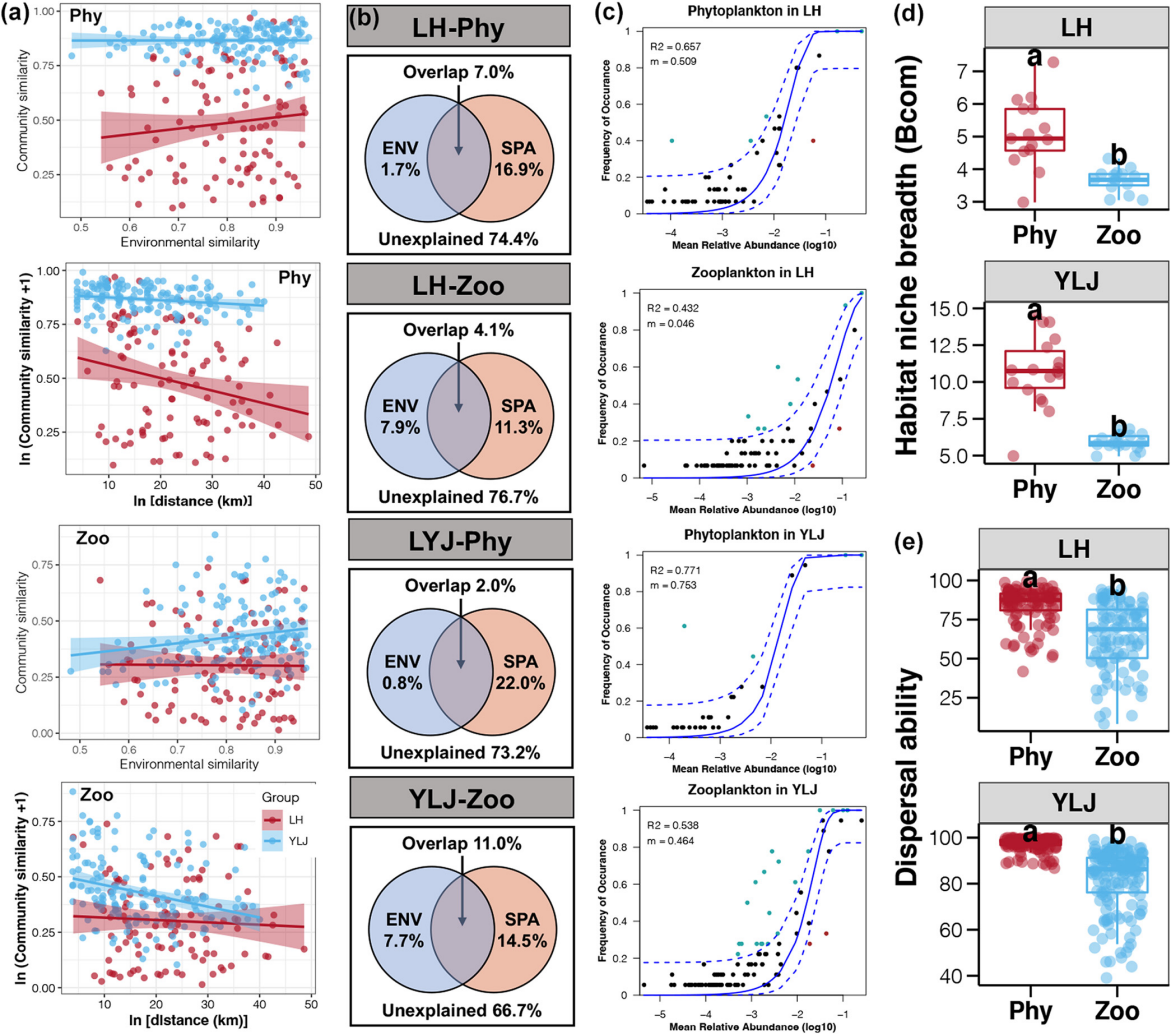
(a) Distance-decay of similarities for phytoplankton and microzooplankton communities in LH and YLJ estuaries based on environmental variables and geographic distances, respectively. (b) VPA of phytoplankton and microzooplankton communities in the LH and YLJ estuaries, respectively, based on environmental (ENV) and spatial (SPA) variables. (c) Fit of the neutral community model of community assembly. The solid blue lines indicate the best fit to the neutral community model and the dashed blue lines represent 95% confidence intervals around the model prediction. ASVs that occur more or less frequently than predicted by the neutral community model are shown in different colors. *m* indicates the immigration of phytoplankton or microzooplankton, *R2* indicates the fit to this model. (d) Mean habitat niche breadth from all taxa in each sample (Bcom) of phytoplankton versus microzooplankton communities in LH and YLJ estuaries, respectively. (e) Dispersal ability of phytoplankton versus microzooplankton communities in the LH and YLJ estuaries, respectively. Different lowercase letters above each box in the same subfigure represent significant differences between groups (*t* test, *P* < 0.05).

Distance-decay of similarity of phytoplankton and microzooplankton communities based on environmental variables and geographic distances were evaluated in LH and YLJ, respectively (Fig. 4a). Significant distance-decay of similarities of geographic distances were found in phytoplankton communities from both LH and YLJ estuaries (Table S4, linear regression, *P* < 0.05). Among them, the slopes of the LH group were higher than that of the YLJ group (Table S4). In contrast, the distance-decay of similarities between environmental variables and phytoplankton communities were not significant (Table S4, linear regression, *P* > 0.05). For microzooplankton communities, both significant environmental and geographic distance-decay similarities were found in YLJ estuaries but not in LH samples (Table S4). Moreover, the power of distance-decay similarity for geographic distance was stronger than that for environmental variables in microzooplankton communities from YLJ (Table S4). VPA was further applied to quantify the relative contributions of environmental selection and passive dispersal on the variations of phytoplankton and microzooplankton communities (Fig. 4b). Regarding variations in phytoplankton communities, the pure effects of spatial variables were stronger in both LH and YLJ estuaries than those of environmental variables (16.9% versus 1.7% in LH and 22.0% versus 0.8% in YLJ). Regarding variations in microzooplankton communities, the pure effect of environmental variables increased; however, it was also lower than that of spatial variables (7.9% versus 11.3% in LH and 7.7% versus 14.5% in YLJ). These results indicated that the phytoplankton and microzooplankton communities in the LH and YLJ estuaries were strongly more governed by passive dispersal than environmental selection.

### Community assembly of phytoplankton and microzooplankton.

To explore the mechanisms responsible for the observed community patterns, neutral community model was performed (Fig. 4c). In the LH and YLJ estuaries, 65.7% and 77.1% phytoplankton ASVs, respectively, can be explained by the neutral community models, indicating that stochastic process dominated the assembly of phytoplankton communities. The explained ratios of neutral community models were much lower for microzooplankton communities (43.2% and 53.8% in LH and YLJ, respectively). These results indicated that deterministic process, such as environmental selection, contributed more to microzooplankton communities. Moreover, the habitat niche breadth and dispersal ability for phytoplankton communities were significantly higher than those for microzooplankton communities in both LH and YLJ estuaries (*t* test, *P* < 0.05, Fig. 4d).

**FIG 5:**
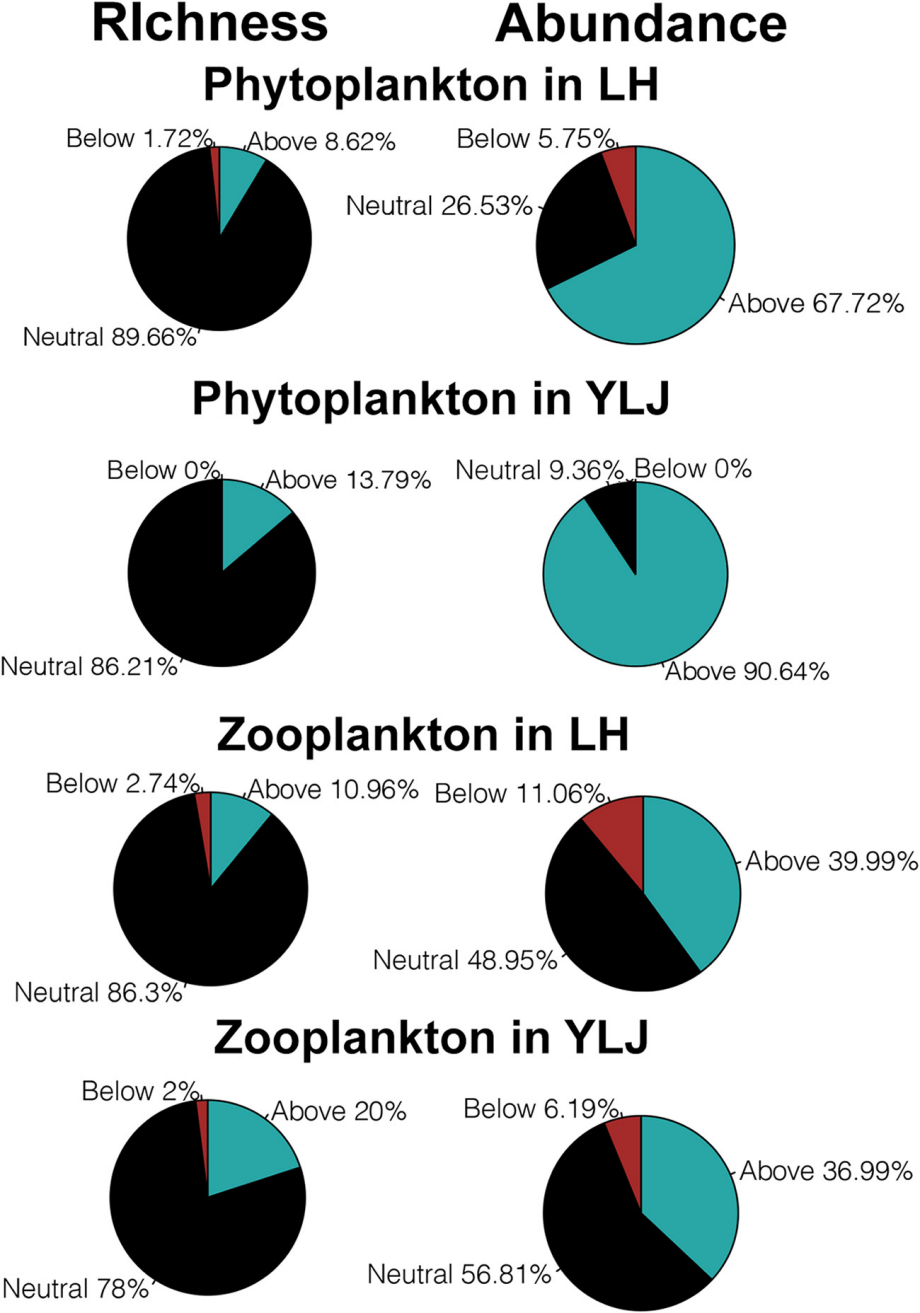
The distinct fitting proportions of phytoplankton and microzooplankton ASVs and sequence numbers by neutral community models.

Phytoplankton and microzooplankton in the LH and YLJ estuaries can be separated into three fractions comprising those ASVs found above, below, and neutral partitions (Fig. 5). The proportions of phytoplankton and microzooplankton ASVs showed that neutral fraction accounted for much higher richness than the above and below fractions in both LH and YLJ estuaries. However, the abundance proportions of phytoplankton ASVs were dominant by the above fraction (67.72% in LH and 90.64% in YLJ). In contrast, although the abundance proportions of microzooplankton ASVs were dominant by the neutral fraction, the abundance proportions for above and below fractions also increased compared to those for richness (Fig. 5).

**FIG 6:**
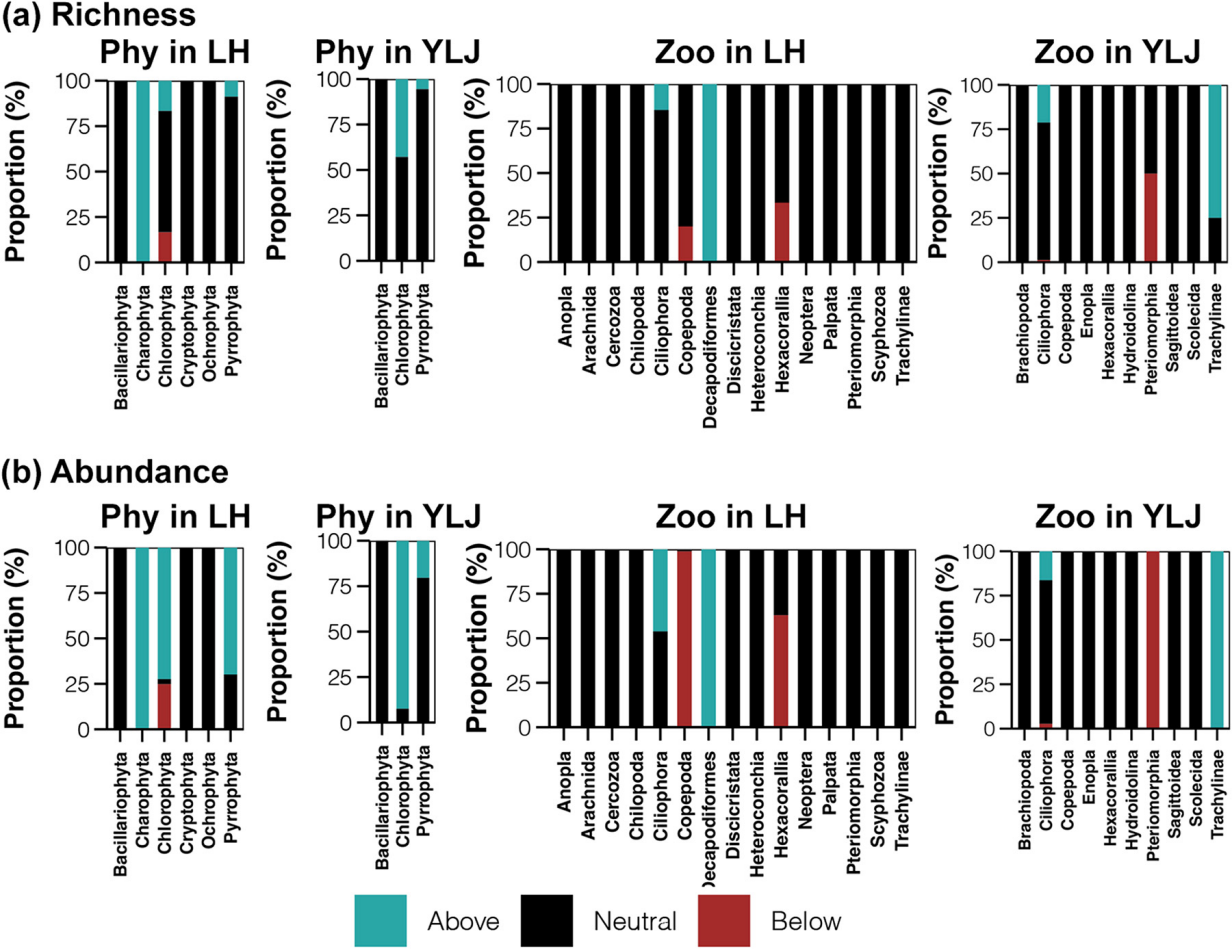
The distinct fitting proportions of richness (ASV number, a) and abundance (sequence number, b) of phytoplankton phyla and microzooplankton lineages in the LH and YLJ estuaries.

Taxa above the prediction are supposed to have higher migration ability and can disperse to more locations. Below the prediction are taxa with lower dispersal ability in the estuaries. For phytoplankton in LH, Charophyta, Chlorophyta, Pyrrophyta occupied the richness of the above fraction (Fig. 6a). In comparison, the abundance proportions of Chlorophyta and Pyrrophyta for the above fraction were notably higher (Fig. 6b), indicating that these two phyla contributed most to the migration of phytoplankton in LH. The same phenomenon was also observed for Chlorophyta in YLJ (Fig. 6). In addition, the abundance proportion of Pyrrophyta in YLJ estuary was also higher than richness proportion of it (Fig. 6). According to the results of microzooplankton, the migration of microzooplankton in the LH and YLJ estuaries were contributed by Ciliophora and Trachylinae, respectively (Fig. 6). Moreover, Copepoda and Hexacorallia in LH as well as Pteriomorphia in YLJ were found to be less dispersible (Fig. 6).

## DISCUSSION

Phytoplankton and microzooplankton are foundational components in aquatic ecosystems where they play a key role in energy flows and biogeochemical cycles. Ecological processes that shape the phytoplankton and microzooplankton communities are among most longstanding issues in microbial ecology. However, the driving factors and assembly mechanisms of sympatric phytoplankton and microzooplankton communities remain poorly understood, especially in vulnerable aquatic ecosystems affected by strong natural and anthropogenic disturbances. Recent development of high-throughput sequencing based on eDNA metabarcoding provides a feasible option for monitoring the microbial communities with fast, standardized, and comprehensive features (29). The sequencing data were used to investigate the diversity, composition, and assembly mechanisms of both phytoplankton and microzooplankton communities in estuarine areas of main rivers in northeast China with remarkable environmental heterogeneity and geographic segregation. Despite the successful application of eDNA metabarcoding technology in the present study, it still has some shortfalls. The lack of a thorough barcode data set to base the eDNA analysis is the biggest obstacle for application the eDNA metabarcoding technology (30), which could lead to false negative and a few wrong taxonomic results (31). In addition, further comparison between the results from classical analysis with the use of microscopes could benefit to evaluate the accuracy of eDNA metabarcoding for microplankton (28).

### Effects of environmental heterogeneity on the phytoplankton and microzooplankton communities in estuarine ecosystems.

According to the results of alpha diversity indices, the richness of phytoplankton was significantly higher in the LH estuary, while that of microzooplankton was higher in YLJ (Fig. 2a). Phytoplankton generally thrives in nutrient-rich environments (29), to which the LH estuary belongs (Fig. S7). Positive correlations between phytoplankton richness and some environmental variables, such as COD and phosphate, observed in this study further confirmed the effects of nutrients on phytoplankton communities (Fig. S8a). Some phytoplankton species can be used as a food source for microzooplankton (32), thus phytoplankton and microzooplankton abundances in the same ecosystem usually show the opposite trends (33). Promotion of microzooplankton can lead to a sustained decrease in phytoplankton, despite high nutrient levels (34–36). Besides, environments with high microzooplankton abundances always had a higher level of ammonia nitrogen due to the limited phytoplankton (37, 38), which can consume almost all the ammonia nitrogen when phytoplankton form blooms (39). This phenomenon was consistent with the higher ammonia concentration and microzooplankton richness in the YLJ estuary compared to the LH samples (Fig. S8b). Moreover, multiple heavy metals were found to be positively correlated with the phytoplankton richness in the LH and YLJ estuaries (Fig. S8). In a previous study at the Bohai Sea, effects of heavy metal pollution on phytoplankton communities and hence resulting in variations of phytoplankton communities had also been proposed (17).

Furthermore, the PCoA illustrated that phytoplankton and microzooplankton communities from the same habitat clustered together (Fig. 2b), indicating distinct community composition from different habitats. Environmental heterogeneity and geographic segregation may explain this phenomenon. First, this result might have resulted from the differences in environmental conditions between different habitats (40). Based on the same samples from this study, we have shown significant environmental heterogeneity between LH and YLJ estuaries (23). Significantly higher concentrations of PHs and multiple heavy metals were detected in the LH estuary compared to the YLJ (*t* test, *P* < 0.05, Fig. S7). In contrast, the YLJ samples possessed significantly higher ammonium concentrations (*t* test, *P* < 0.05, Fig. S7). In addition, water temperature and salinity were two and six degrees lower in the YLJ estuary compared to LH (*t* test, *P* < 0.05, Fig. S7). Second, geographical segregation could result in the presence of regional species pool and hence different community composition (41). Only 14.5% phytoplankton ASVs and 16.1% microzooplankton ASVs were found to be shared between the LH and YLJ estuaries (Fig. S9), revealing huge differences in regional species pools between these two habitats.

### Passive dispersal shaping phytoplankton community but environmental selection shaping microzooplankton community.

Environmental and spatial factors are two major factors to influence the community assembly in natural environments (42). In the present study, several environmental variables were identified as having a significant effect on the phytoplankton and microzooplankton compositions by canonical correlation analysis (Table S5). Meanwhile, significant geographic distance-decay of similarities for phytoplankton and microzooplankton communities were also observed in both LH and YLJ estuaries (Fig. 4a). Although significant effects of environmental and spatial factors on the phytoplankton and microzooplankton communities were uncovered, results of VPA in this study indicated that most of the community variation remained unexplained by them in both estuaries (Fig. 4b). VPA is widely used in ecological research to determine the contribution of different factors on community variations; however, it will underestimate the role of environmental factors and should be applied together with other approaches (43). In this study, the neutral community model was used to adjust VPA and infer the influences of ecological processes.

Our results clearly support the prominent role of stochastic processes in shaping the phytoplankton community assembly of both LH and YLJ estuaries (Fig. 4c). According to the ecological theory, stochastic processes shaping the community assembly can be divided into dispersal and ecological drift (44). Based on the neutral theory, community similarity was predicted to decrease along spatial distances due to dispersal limitation when stochastic processes govern the community assembly (45). The significant and strong distance-decay pattern of phytoplankton communities in the LH and YLJ estuaries is indicative that dispersal was important (Fig. 4a). Comparatively, the relative importance of stochastic processes to shape the microzooplankton communities was much lower than that for phytoplankton based on the neutral community models (Fig. 4c). These results suggest deterministic selection by environmental factors shaped the microzooplankton communities due to the preferences and fitness of taxa (46), which were consistent with the higher contribution of environmental variables to microzooplankton communities shown by distance-decay of similarity and VPA (Fig. 4a and b). Regarding the community immigration rate, the *m* values of neutral community models for phytoplankton in the LH and YLJ estuaries were higher than the microzooplankton (0.509 versus 0.054 in LH and 0.753 versus 0.464 in YLJ, Fig. 4c). These results were consistent with the average shared proportion of taxa in the LH and YLJ estuaries (Fig. 4e) and indicated the higher dispersal ability of phytoplankton than microzooplankton. Moreover, wider niche breadth means more metabolic adaptation and less environmental selection for species in a community (47). Significantly wider niche breadth of phytoplankton than microzooplankton in LH and YLJ estuaries further confirmed the importance of environmental selection on microzooplankton community assembly (Fig. 4d). Token together, our results revealed that passive dispersal shaping phytoplankton community but environmental selection shaping microzooplankton community in estuarine ecosystems.

### Difference between neutral and nonneutral partitions.

The neutral community model separated taxa into neutral and nonneutral partitions, which were possibly attributable to the different dispersal rates among taxa (43). Chlorophyta had the highest dispersal ability in phytoplankton communities from both the LH and YLJ estuaries (Fig. 6), which was also more abundant in YLJ estuary (Fig. 3b). Chlorophyta has been reported to have higher dispersal ability because its spores and resistant vegetative fragments facilitate dispersion and desiccation tolerance (48). In addition, higher ammonia concentrations in YLJ could be the reason for Chlorophyta to bloom in this area (49). In addition, Pyrrophyta were more abundant in LH and played a vital role in phytoplankton dispersal (Fig. 3b and 6). Pyrrophyta typically have two flagella that allow cells to move actively (50). Relative water temperature and salinity in the LH estuary could be the reason of higher abundances of Pyrrophyta (51). Besides, petroleum pollution, one of the main contaminations in LH estuary, has been shown to cause the initiation of Pyrrophyta blooms (52). For microzooplankton, Ciliophora were found to be the main mobile microzooplankton in the LH estuary (Fig. 6), which were also more abundant in LH estuary compared to YLJ samples (Fig. 3e). In accordance with our results, previous studies have revealed that Ciliophora were positively correlated with concentrations of nutrients (53, 54). These suggest that Ciliophora in estuaries could actively move to locations with high nutrients. Trachylinae was the microzooplankton lineage with the highest degree of dispersal in the YLJ estuary (Fig. 6). At the landward side of the YLJ estuary, many aquaculture ponds are in operation, and jellyfish, a subclass of Trachylinae, is one of the main cultivated organisms (55). Larvae entering the estuarine area through water exchange may be the reason for the higher abundance of Trachylinae in the YLJ estuary. At the same time, jellyfish is a highly mobile taxa and can move to areas farther offshore (56).

### Conclusions.

Our study demonstrated the feasibility of eDNA meabarcoding sequencing to simultaneously capture the diversity and composition of phytoplankton and microzooplankton in the aquatic environments. Based on the diversity comparisons for the LH and YLJ estuaries, richness of phytoplankton was significantly higher in LH than in YLJ, while microzooplankton richness was higher in YLJ. Remarkable differences of phytoplankton and microzooplankton compositions between the LH and YLJ estuaries were observed, which could be due to environmental heterogeneity and geographic segregation between these two regions. Neutral community models showed that stochastic processes dominated the phytoplankton community assembly in both estuaries. In contrast, the relative importance of stochastic processes in microzooplankton community assembly was relatively low. Furthermore, results of distance-decay similarity and VPA indicated that passive dispersal shaped phytoplankton community but environmental selection shaped microzooplankton community in the two estuaries. Phytoplankton and microzooplankton taxa that did not conform to the neutral distribution were found in LH and YLJ estuaries, which usually have active mobility and might be selected by local environmental conditions. Based on the investigation of model estuarine systems, our results reveal the differences in planktonic communities between estuaries at similar latitudes with similar climate conditions but with different geomorphologies and anthropogenic impacts. Furthermore, our in-depth ecological model analyses shed lights on the underlying assembly mechanisms of planktonic communities, highlighting the importance of environmental heterogeneity and geographic segregation on planktonic community assemblages.

## MATERIALS AND METHODS

### Sample collection and measurements of environmental variables.

A total of 15 and 18 surface water samples were collected from LH and YLJ estuaries, respectively, in July 2019 (Fig. 1a). For each sample, approximately 2 L of surface water (~0.5 m depth under the water) was collected with a water sampler (Wuhan Shuitiandi Instruments, Wuhan, China) and temporarily held on ice for less than 6 h before filtration. For DNA samples, 1 L of each sample was filtered through 0.2-μm pore size polycarbonate membranes (142 mm diameter, Millipore, USA) with a peristaltic pump. The filters were immediately preserved in liquid nitrogen, transported to laboratory, and stored at −80°C until DNA extraction. The remaining 1 L of water was placed in a clean and sterile glass container for environmental variables analyses in the laboratory. A total of 15 environmental variables, including water temperature (Temp), salinity, dissolved oxygen (DO), chemical oxygen demand (COD), petroleum hydrocarbons (PHs), nitrate, nitrite, ammonium, phosphate, and six heavy metals (Cu, Pb, Zn, Cd, Cr, and As) were measured at each sampling station as previously described (23).

### DNA extraction and sequencing.

Total DNA for each water sample was extracted from the filters using a PowerWater DNA isolation kit (Qiagen, CA, USA) according to the manufacturer's instructions. Agarose gel electrophoresis (1% concentration) was used to detect the success of DNA extraction. DNA qualities were measured using a NanoDrop ND-1000 Spectrophotometer (NanoDrop, USA). The 18S rRNA V9 region from each extracted DNA were amplified using primers 1380F-1510R (1380F: TCCCTGCCHTTTG TACACAC, 1510R: CCTTCYGCAGGTTCACCTAC) (57). PCR amplification and sequencing were done as previously described (23). Raw data have been deposited to the US National Center for Biotechnology Information (NCBI) under the BioProject number PRJNA782015.

### Data processing.

Reads with an average Phred score (Q score) lower than 20, ambiguous bases, a homopolymer greater than 6, and mismatches in primers were deleted (58). Remaining high-quality reads were ascribed to the samples based on their unique barcodes at the end of reverse primers. These reads were then assigned to amplicon sequence variants (ASVs) with the Quantitative Insights Into Microbial Ecology 2 (QIIME2) through the Divisive Amplicon Denoising Algorithm 2 (DADA2) method (59). Singletons (number of a specific ASV = 1) were abandoned to improve the efficiency of data analysis. Taxonomic annotation of ASVs was performed using the QIIME2 pipeline based on the SILVA 138 database (60). ASVs belonging to phytoplankton and microzooplankton were binned separately using an in-house R script, and then the taxonomic names at phylum level were rematched. Finally, the phytoplankton and microzooplankton ASV abundance tables were normalized using a standard number of reads according to the sample with the least number of reads (5,897 and 3,966 tags for phytoplankton and microzooplankton, respectively).

### Statistical analysis.

All statistics analyses were performed using R v4.0.2 platform. Rarefaction and species cumulative curves of sequenced data sets were extrapolated by “iNEXT” package. Four alpha diversity indices of phytoplankton and microzooplankton communities, including Chao1, Shannon, Pielou_J, and Pd_faith, were calculated by “vegan” package. Variations in the composition of phytoplankton and microzooplankton were evaluated by principal coordinate analysis (PCoA) and the Adonis test based on the Bray-Curtis distance using the “vegan” package. Differences in alpha diversity indices, and community distances between samples from LH and YLJ estuaries were analyzed using a Student's *t* test (t.test function). Differences in relative abundances of phytoplankton and microzooplankton taxa between LH and YLJ estuaries were analyzed using the Wilcox Rank-Sum test (wilcox.test function). Results of differentially abundant taxa and relative abundances of dominant taxa of phytoplankton and microzooplankton communities were visualized by “ggplot2” package. Furthermore, Random Forest analyses were performed using the “RandomForest” package to recognize key phytoplankton and microzooplankton genera for distinguishing the origins of samples. The relative abundances of these key taxa and the accuracy of the Random Forest model were displayed using the “ggplot2” and “pheatmap” packages, respectively.

Euclidean distance of environmental variables (“vegan” package) and geographic distance (“geosphere” package) between any two sampling stations were calculated, and then linear regression (lm function) was applied to evaluate the distance-decay of similarities of phytoplankton and microzooplankton communities. In addition, variation partitioning analysis (VPA) was completed by “vegan” package to determine the relative importance of environmental and spatial factors in shaping phytoplankton and microzooplankton communities in the LH and YLJ estuaries. Moreover, a neutral community model was used to determine the potential importance of stochastic process on assembly of phytoplankton and microzooplankton communities. In this model *m* is an estimate of dispersal between communities and *R2* represents the ratio of contribution by stochastic processes (61). The ASVs from each data set were subsequently separated into three partitions depending on whether they occurred more frequently than (above partition), less frequently than (below partition), or within (neutral partition) the 95% confidence interval of the neutral community model predictions. The richness and composition of these separated partitions were further compared, and the results were displayed by “ggplot2” package.

Furthermore, niche breadth and dispersal ability are critical traits that influence the relative importance of environmental selection and passive dispersal (47, 62). Niche breadth of phytoplankton and microzooplankton communities in the LH and YLJ estuaries was measured by the “spaa” package based on Levins' niche breadth index. The average B-values from all taxa in a single community (Bcom) as an indicator of habitat niche breadth at the community level. A wider niche breadth of an organism group means more metabolically flexible at the community level (47). To estimate dispersal ability of each taxon (passively driven by water movements), we calculated the pairwise shared proportion of sequence numbers, and used the average shared proportion as a proxy for dispersal. A higher shared proportion of sequences indicates a more successful passive transport by currents (63). The Student's *t* test was performed to evaluate the differences in niche breadth and dispersal ability between the phytoplankton and microzooplankton communities in the LH and YLJ estuaries, respectively.

### Data availability.

All raw sequences of sediment bacterial communities studied in this study have been submitted to the NCBI Sequence Read Archive (SRA) database under the BioProject number PRJNA782015.

## References

[1] Lu Z, Na G, Gao H, Wang L, Bao C, Yao Z. 2015. Fate of sulfonamide resistance genes in estuary environment and effect of anthropogenic activities. Sci Total Environ 527–528:429–438. doi:10.1016/j.scitotenv.2015.04.101. 25981941

[2] Liu JY. 2013. Status of marine biodiversity of the China seas. PLoS One 8:e50719. doi:10.1371/journal.pone.0050719. 23320065 PMC3540058

[3] Costanza R, d'Arge R, de Groot R, Farber S, Grasso M, Hannon B, Limburg K, Naeem S, O'Neill RV, Paruelo J, Raskin RG, Sutton P, van den Belt M. 1997. The value of the world's ecosystem services and natural capital. Nature 387:253–260. doi:10.1038/387253a0.

[4] Lotze HK. 2010. Historical reconstruction of human-induced changes in U.S. estuaries. Oceanogr Mar Biol 48:267–338.

[5] Elliott M, Whitfield AK. 2011. Challenging paradigms in estuarine ecology and management. Estuar Coast Shelf S 94:306–314. doi:10.1016/j.ecss.2011.06.016.

[6] Samuli K, Andersen JH. 2016. A global review of cumulative pressure and impact assessments in marine environment. Front Mar Sci 3:10–3389.

[7] Nemergut DR, Schmidt SK, Fukami T, O'Neill SP, Bilinski TM, Stanish LF, Knelman JE, Darcy JL, Lynch RC, Wickey P, Ferrenberg S. 2013. Patterns and processes of microbial community assembly. Microbiol Mol Biol Rev 77:342–356. doi:10.1128/MMBR.00051-12. 24006468 PMC3811611

[8] Hanson CA, Fuhrman JA, Horner-Devine MC, Martiny JBH. 2012. Beyond biogeographic patterns: processes shaping the microbial landscape. Nat Rev Microbiol 10:497–506. doi:10.1038/nrmicro2795. 22580365

[9] Szekely AJ, Silke L. 2014. The importance of species sorting differs between habitat generalists and specialists in bacterial communities. FEMS Microbiol Ecol 1:102–112. 10.1111/1574-6941.12195 23991811

[10] Chen L, Liu S, Chen Q, Zhu G, Wu X, Wang J, Li X, Hou L, Ni J. 2020. Dispersal limitation drives biogeographical patterns of anammox bacterial communities across the Yangtze River. Appl Microbiol Biotechnol 104:5535–5546. doi:10.1007/s00253-020-10511-4. 32300854

[11] Zamora-Terol S, Novotny A, Winder M. 2020. Reconstructing marine plankton food web interactions using DNA metabarcoding. Mol Ecol 29:3380–3395. doi:10.1111/mec.15555. 32681684

[12] Roelke DL, Li HP, Miller–DeBoer CJ, Gable GM, Davis SE. 2017. Regional shifts in phytoplankton succession and primary productivity in the San Antonio Bay System (USA) in response to diminished freshwater inflows. Mar Freshw Res 68:131–145. doi:10.1071/MF15223.

[13] Sun XM, Steinman AD, Xue QJ, Zhao YY, Tang XM, Xie LQ. 2017. Temporal patterns of phyto- and bacterio-plankton and their relationships with environmental factors in Lake Taihu, China. Chemosphere 184:299–308. 28601663 10.1016/j.chemosphere.2017.06.003

[14] Liu S, Cui Z, Zhao Y, Chen N. 2022. Composition and spatial-temporal dynamics of phytoplankton community shaped by environmental selection and interactions in the Jiaozhou Bay. Water Res 218:118488. doi:10.1016/j.watres.2022.118488. 35489150

[15] Shayestehfar A, Noori M, Shirazi F. 2010. Environmental factor effects on the seasonally changes density in Parishan lake (Khajoo Spring site) Iran. Asian J Exp Biol Sci 1:840–844.

[16] Li C, Feng W, Chen H, Li X, Song F, Guo W, Giesy JP, Sun F. 2019. Temporal variation in microzooplankton and phytoplankton community species composition and the affecting factors in Lake Taihud–a large freshwater lake in China. Environ Pollut 245:1050–1057. doi:10.1016/j.envpol.2018.11.007. 30682739

[17] Zhao Z, Li H, Sun Y, Yang Q, Fan J. 2021. Contrasting the assembly of phytoplankton and microzooplankton communities in a polluted semi-closed sea: effects of marine compartments and environmental selection. Environ Pollut 285:117256. doi:10.1016/j.envpol.2021.117256. 33957514

[18] Jones GP, Srinivasan M, Almany GR. 2007. Population connectivity and conservation of marine biodiversity. Oceanog 20:100–111. doi:10.5670/oceanog.2007.33.

[19] Spatharis S, Lamprinou V, Meziti A, Kormas KA, Danielidis DD, Smeti E, Roelke DL, Mancy R, Tsirtsis G. 2019. Everything is not everywhere: can marine compartments shape phytoplankton assemblages? Proc R Soc B 286:20191890. doi:10.1098/rspb.2019.1890. PMC6842854 31662088

[20] Ministry of Ecology and Environment. 2018. The People’s Republic of China. Bulletin of marine ecology and environment status of China in 2018. https://english.mee.gov.cn/Resources/Reports/bomeaesoc/201911/P020191129369234962072.pdf.

[21] Li H, Lin L, Ye S, Li H, Fan J. 2017. Assessment of nutrient and heavy metal contamination in the seawater and sediment of Yalujiang Estuary. Mar Pollut Bull 117:499–506. doi:10.1016/j.marpolbul.2017.01.069. 28185654

[22] Sun Y, Li H, Yang Q, Liu Y, Fan J, Guo H. 2020. Disentangling effects of river inflow and marine diffusion in shaping the planktonic communities in a heavily polluted estuary. Environ Pollut 267:115414. doi:10.1016/j.envpol.2020.115414. 33254723

[23] Zhao Z, Li H, Sun Y, Shao K, Wang X, Ma X, Hu A, Zhang H, Fan J. 2022. How habitat heterogeneity shapes bacterial and protistan communities in temperate coastal areas near estuaries. Environ Microbial 24:1775–1789. doi:10.1111/1462-2920.15892. 34996132

[24] Zhang K, Jiang F, Chen H, Dibar DT, Wu Q, Zhou Z. 2019. Temporal and spatial variations in microzooplankton communities in relation to environmental factors in four floodplain lakes located in the middle reach of the Yangtze River, China. Environ Pollut 251:277–284. doi:10.1016/j.envpol.2019.04.139. 31082612

[25] Pawlowski J, Kelly-Quinn M, Altermatt F, Apotheloz-Perret-Gentil L, Beja P, Boggero A, Borja A, Bouchez A, Cordier T, Domaizon I. 2018. The future of biotic indices in the ecogenomic era: integrating (e)DNA metabarcoding in biological assessment of aquatic ecosystems. Sci Total Environ 637:1295–1310. 29801222 10.1016/j.scitotenv.2018.05.002

[26] Sagova-Mareckova M, Boenigk J, Bouchez A, Cermakova K, Chonova T, Cordier T, Eisendle U, Elersek T, Fazi S, Fleituch T, Frühe L, Gajdosova M, Graupner N, Haegerbaeumer A, Kelly A-M, Kopecky J, Leese F, Nõges P, Orlic S, Panksep K, Pawlowski J, Petrusek A, Piggott JJ, Rusch JC, Salis R, Schenk J, Simek K, Stovicek A, Strand DA, Vasquez MI, Vrålstad T, Zlatkovic S, Zupancic M, Stoeck T. 2021. Expanding ecological assessment by integrating microorganisms into routine freshwater biomonitoring. Water Res 191:116767. doi:10.1016/j.watres.2020.116767. 33418487

[27] Djurhuus A, Pitz K, Sawaya NA, Rojas-Márquez J, Michaud B, Montes E, Muller-Karger F, Breitbart M. 2018. Evaluation of marine microzooplankton community structure through environmental DNA metabarcoding. Limnology & Ocean Methods 16:209–221. doi:10.1002/lom3.10237. PMC5993268 29937700

[28] Fan J, Wang S, Li H, Yan Z, Zhang Y, Zheng X, Wang P. 2020. Modeling the ecological status response of rivers to multiple stressors using machine learning: a comparison of environmental DNA metabarcoding and morphological data. Water Res 183:116004. doi:10.1016/j.watres.2020.116004. 32622231

[29] Basu S, Mackey KR. 2018. Phytoplankton as key mediators of the biological carbon pump: their responses to a changing climate. Sustainability 10:869. doi:10.3390/su10030869.

[30] Zhong W, Zhang J, Wang Z, Lin J, Huang X, Liu W, Li H, Pellissier L, Zhang X. 2022. Holistic Impact Evaluation of Human Activities on the Coastal Fish Biodiversity in the Chinese Coastal Environment. Environ Sci Technol 56:6574–6583. doi:10.1021/acs.est.2c01339. 35510674

[31] Zhang Z, Li J, Li H, Wang L, Zhou Y, Li S, Zhang Z, Feng K, Deng Y. 2023. Environmental DNA metabarcoding reveals the influence of human activities on microeukaryotic plankton along the Chinese coastline. Water Res 233:119730. doi:10.1016/j.watres.2023.119730. 36801577

[32] Yong YL, Lee CW, Bong CW, Chew LL, Chong VC. 2022. The role of microzooplankton grazing in the microbial food web of a tropical mangrove estuary. Estuar Coast Shelf S 275:107969. doi:10.1016/j.ecss.2022.107969.

[33] Irigoien X, Huisman J, Harris RP. 2004. Global biodiversity patterns of marine phytoplankton and microzooplankton. Nature 429:863–867. doi:10.1038/nature02593. 15215862

[34] Belfiore AP, Buley RP, Fernandez-Figueroa EG, Gladfelter MF, Wilson AE. 2021. Microzooplankton as an alternative method for controlling phytoplankton in catfish pond aquaculture. Aquacult Rep 21:100897. doi:10.1016/j.aqrep.2021.100897.

[35] Burkill PH, Mantoura RFC, Llewellyn CA, Owens NJP. 1987. Microzooplankton grazing and selectivity of phytoplankton in coastal waters. Mar Biol 93:581–590. doi:10.1007/BF00392796.

[36] Caron DA, Hutchins DA. 2013. The effects of changing climate on microzooplankton grazing and community structure: drivers, predictions and knowledge gaps. J Plankton Res 35:235–252. doi:10.1093/plankt/fbs091.

[37] Butts TJ, Moody EK, Wilkinson GM. 2022. Contribution of zooplankton nutrient recycling and effects on phytoplankton size structure in a hypereutrophic reservoir. J Plankton Res 44:839–853. doi:10.1093/plankt/fbac045.

[38] Karakuş O, Völker C, Iversen M, Hagen W, Hauck J. 2022. The role of zooplankton grazing and nutrient recycling for global ocean biogeochemistry and phytoplankton phenology. J Geophys Res Biogeo 127:e2022JG006798. doi:10.1029/2022JG006798.

[39] Suslov SV, Gruzdev VS, Khrustaleva MA, Boitsenyuc LI. 2020. The effects of climate change on the development of phytoplankton in the Uchinsk Reservoir of the Moscow Canal. IOP Conference Series: Earth and Environmental Science 579:012039. IOP Publishing, Philadelphia, PA.

[40] Nagai S, Hida K, Urusizaki S, Takano Y, Hongo Y, Kameda T, Abe K. 2016. Massively parallel sequencing-based survey of eukaryotic community structures in Hiroshima Bay and Ishigaki Island. Gene 576:681–689. doi:10.1016/j.gene.2015.10.026. 26476293

[41] Kraft NJB, Comita LS, Chase JM, Sanders NJ, Swenson NG, Crist TO, Stegen JC, Vellend M, Boyle B, Anderson MJ, Cornell HV, Davies KF, Freestone AL, Inouye BD, Harrison SP, Myers JA. 2011. Disentangling the drivers of β diversity along latitudinal and elevational gradients. Science 333:1755–1758. doi:10.1126/science.1208584. 21940897

[42] Nagai S, Hida K, Urushizaki S, Onitsuka G, Yasuike M, Nakamura Y, Fujiwara A, Tajimi S, Kimoto K, Kobayashi T, Gojobori T, Ototake M. 2015. Influences of diurnal sampling bias on fixed-point monitoring of plankton biodiversity determined using a massively parallel sequencing-based technique. Gene:667–675. 10.1016/j.gene.2015.10.025 26475937

[43] Chen W, Ren K, Isabwe A, Chen H, Liu M, Yang J. 2019. Stochastic processes shape microeukaryotic community assembly in a subtropical river across wet and dry seasons. Microbiome 7:1–16. 31640783 10.1186/s40168-019-0749-8 PMC6806580

[44] Tong X, Leung MHY, Wilkins D, Cheung HHL, Lee PKH. 2019. Neutral processes drive seasonal assembly of the skin mycobiome. mSystems 4:e00004-19. doi:10.1128/mSystems.00004-19. 30944878 PMC6435813

[45] Hubbell SP. 2001. A unified neutral theory of biodiversity and biogeography. Princeton University Press, Princeton, NJ.

[46] Zhou JZ, Ning DL. 2017. Stochastic community assembly: does it matter in microbial ecology? Microbiol Mol Biol Rev 81:e00002-17. doi:10.1128/MMBR.00002-17. 29021219 PMC5706748

[47] Pandit SN, Kolasa J, Cottenie K. 2009. Contrasts between habitat generalists and specialists: an empirical extension to the basic metacommunity framework. Ecology 90:2253–2262. doi:10.1890/08-0851.1. 19739387

[48] Rodríguez-Flores R, Carmona-Jiménez J. 2018. Ecology and distribution of macroscopic algae communities in streams from the Basin of Mexico. Bot Sci 96:63–75. doi:10.17129/botsci.1237.

[49] Dai GZ, Shang JL, Qiu BS. 2012. Ammonia may play an important role in the succession of cyanobacterial blooms and the distribution of common algal species in shallow freshwater lakes. Glob Change Biol 18:1571–1581. doi:10.1111/j.1365-2486.2012.02638.x.

[50] Taylor FJR, Hoppenrath M, Saldarriaga JF. 2008. Dinoflagellate diversity and distribution. Biodivers Conserv 17:407–418. doi:10.1007/s10531-007-9258-3.

[51] Alkawri AAS, Ramaiah N. 2010. Spatio-temporal variability of dinoflagellate assemblages in different salinity regimes in the west coast of India. Harmful Algae 9:153–162. doi:10.1016/j.hal.2009.08.012.

[52] Almeda R, Cosgrove S, Buskey EJ. 2018. Oil spills and dispersants can cause the initiation of potentially harmful dinoflagellate blooms (“Red Tides”). Environ Sci Technol 52:5718–5724. doi:10.1021/acs.est.8b00335. 29659258

[53] Hou FR, Zhang HJ, Xie WJ, Zhou XY, Zhu XY, Zhang DM. 2020. Co-occurrence patterns and assembly processes of microeukaryotic communities in an early-spring diatom bloom. Sci Total Environ 711:134624. doi:10.1016/j.scitotenv.2019.134624. 31818596

[54] Zou K, Wang R, Xu S, Li Z, Liu L, Li M, Zhou L. 2021. Changes in protist communities in drainages across the Pearl River Delta under anthropogenic influence. Water Res 200:117294. doi:10.1016/j.watres.2021.117294. 34102388

[55] Choi CY, Battley PF, Potter MA, Ma Z, Liu W. 2014. Factors affecting the distribution patterns of benthic invertebrates at a major shorebird staging site in the Yellow Sea. China Wetlands 34:1085–1096. doi:10.1007/s13157-014-0568-4.

[56] Purcell JE, Uye SI, Lo WT. 2007. Anthropogenic causes of jellyfish blooms and their direct consequences for humans: a review. Mar Ecol Prog Ser 350:153–174. doi:10.3354/meps07093.

[57] Amaral-Zettler LA, McCliment EA, Ducklow HW, Huse SM. 2009. A method for studying protistan diversity using massively parallel sequencing of V9 hypervariable regions of small-subunit ribosomal RNA genes. PLoS One 4:e6372. doi:10.1371/journal.pone.0006372. 19633714 PMC2711349

[58] Bokulich NA, Subramanian S, Faith JJ, Gevers D, Gordon JI, Knight R, Mills DA, Caporaso JG. 2013. Quality-filtering vastly improves diversity estimates from Illumina amplicon sequencing. Nat Methods 10:57–59. doi:10.1038/nmeth.2276. 23202435 PMC3531572

[59] Bokulich NA, Kaehler BD, Rideout JR, Dillon M, Bolyen E, Knight R, Huttley GA, Caporaso JG. 2018. Optimizing taxonomic classification of marker-gene amplicon sequences with qiime 2’s q2-feature-classifier plugin. Microbiome 6:90. doi:10.1186/s40168-018-0470-z. 29773078 PMC5956843

[60] Yilmaz P, Parfrey LW, Yarza P, Gerken J, Pruesse E, Quast C, Schweer T, Peplies J, Ludwig W, Glockner FO. 2014. The SILVA and “all-species living tree project (LTOP).” Taxonomic frameworks. Nucleic Acids Res 42:D643–D648. doi:10.1093/nar/gkt1209. 24293649 PMC3965112

[61] Sloan WT, Lunn M, Woodcock S, Head IM, Nee S, Curtis TP. 2006. Quantifying the roles of immigration and chance in shaping prokaryote community structure. Environ Microbiol 8:732–740. doi:10.1111/j.1462-2920.2005.00956.x. 16584484

[62] Bie T, Meester L, Brendonck L, Martens K, Goddeeris B, Ercken D, Hampel H, Denys L, Vanhecke L, Gucht K, Wichelen J, Vyverman W, Declerck SAJ. 2012. Body size and dispersal mode as key traits determining metacommunity structure of aquatic organisms. Ecol Lett 15:740–747. doi:10.1111/j.1461-0248.2012.01794.x. 22583795

[63] Yeh Y-C, Peres-Neto PR, Huang S-W, Lai Y-C, Tu C-Y, Shiah F-K, Gon G-C, Hsieh C-H. 2015. Determinism of bacterial metacommunity dynamics in the southern East China Sea varies depending on hydrography. Ecography 38:198–212. doi:10.1111/ecog.00986.

