# Supplementary material for: Phytoplankton and Microzooplankton Community Structure and Assembly Mechanisms in Northwestern Pacific Ocean Estuaries with Environmental Heterogeneity and Geographic Segregation
Source: Microbiol Spectr. 2023 Mar 20;11(2):e04926-22. doi: 10.1128/spectrum.04926-22 (PMC10100884; doi:10.1128/spectrum.04926-22)
Supplement: Supplemental file 1 — Supplemental material. Download spectrum.04926-22-s0001.pdf, PDF file, 2.0 MB [file spectrum.04926-22-s0001.pdf]

Supporting Information for

Phytoplankton and microzooplankton community structure and assembly mechanisms in Northwestern Pacific Ocean estuaries with environmental heterogeneity and geographic segregation

Yi Sun[1], Hongjun Li[#,1], Xiaocheng Wang[1], Yuan Jin[1], Satoshi Nagai[2], Senjie Lin[3]

[1] State Environmental Protection Key Laboratory of Coastal Ecosystem, National Marine Environmental Monitoring Center, Dalian, 116023, China

[2] Coastal and Inland Fisheries Ecosystems Division, Fisheries Technology Institute, Japan Fisheries Research and Education Agency, Kanagawa 236-8648, Japan

[3] Department of Marine Sciences, University of Connecticut, Groton, CT, United States

[#] Corresponding author. E-mail address: hjli@nmemc.org.cn (H. Li)

Table S1. Statistics of sequenced data.

Sample ID	Input	Filtered	Denoised	Merged	Non-chimeric	Non-singleton	Effective reads of phy	Effective read of zoo
LH1	124,529	117,556	116,857	105,035	104,656	104,656	9,059	10,206
LH2	121,245	113,535	112,400	95,845	95,067	95,067	10,793	15,633
LH3	126,990	119,274	118,139	104,580	103,829	103,829	12,548	19,758
LH4	112,527	105,885	105,226	96,863	96,068	96,068	6,744	26,872
LH5	125,467	117,370	116,553	105,585	105,520	105,520	10,295	14,620
LH6	141,619	130,604	129,683	114,396	114,079	114,079	9,319	18,650
LH7	142,273	134,630	133,781	119,779	119,287	119,286	12,138	14,521
LH8	136,543	126,048	125,494	111,968	110,632	110,632	13,692	14,453
LH9	120,695	111,389	110,775	94,984	94,480	94,479	14,132	1,908
LH10	115,601	107,963	107,350	95,751	94,869	94,869	30,403	2,291
LH11	135,277	127,432	126,708	115,015	114,960	114,960	10,383	17,179
LH12	129,002	121,792	121,247	106,384	106,339	106,338	9,921	15,264
LH13	122,443	115,148	114,506	100,037	99,822	99,822	8,616	20,782
LH14	126,219	118,371	117,650	108,880	107,990	107,990	22,692	7,752
LH15	125,783	118,435	117,777	106,467	105,086	105,086	33,809	4,047
YLJ1	114,376	107,924	107,189	100,054	99,049	99,049	17,637	25,311
YLJ2	111,794	103,215	101,072	93,527	93,132	93,132	16,649	21,875
YLJ3	109,605	101,259	100,665	96,398	94,060	94,060	20,672	24,755
YLJ4	104,433	97,048	96,826	90,117	88,073	88,073	19,349	37,101
YLJ5	102,082	95,692	95,064	88,806	86,339	86,339	20,022	32,229
YLJ6	121,304	113,525	113,220	106,663	106,262	106,262	22,298	33,147
YLJ7	116,213	108,323	107,717	102,011	100,060	100,060	16,003	29,441
YLJ8	104,761	96,918	96,358	89,422	86,877	86,876	15,346	13,673
YLJ9	103,120	95,876	95,034	84,443	81,895	81,895	24,815	16,683
YLJ10	109,060	102,185	101,810	95,302	93,678	93,678	9,118	43,940
YLJ11	139,157	128,938	127,518	117,108	115,287	115,286	5,897	11,789
YLJ12	92,896	86,537	86,268	82,112	79,808	79,808	6,502	3,966
YLJ13	107,750	100,960	100,516	94,677	92,156	92,156	14,399	35,253
YLJ14	108,312	89,858	89,317	86,435	85,159	85,159	15,461	44,845
YLJ15	113,074	106,371	105,984	99,801	98,399	98,399	16,592	32,612
YLJ16	102,775	97,481	96,802	92,171	90,442	90,442	13,343	9,375
YLJ17	100,090	94,778	94,381	90,313	89,362	89,362	17,600	39,902
YLJ18	114,350	108,721	108,308	103,481	102,125	102,125	17,388	32,662

Table S2. Statistics of taxonomy annotation of phytoplankton in Liaohe and Yalujiang estuaries.

Taxonomy	Phylum	Family	Genus	Species
LH1	3	7	7	2
LH2	3	10	11	3
LH3	4	7	9	3
LH4	2	4	4	2
LH5	3	5	7	2
LH6	4	6	4	2
LH7	5	10	10	4
LH8	3	6	7	4
LH9	4	8	6	1
LH10	3	6	3	1
LH11	2	4	4	2
LH12	3	7	7	3
LH13	4	8	9	2
LH14	2	3	1	0
LH15	4	8	7	3
YLJ1	2	3	5	2
YLJ2	3	4	6	1
YLJ3	2	3	5	2
YLJ4	3	4	6	1
YLJ5	3	5	7	2
YLJ6	2	2	4	1
YLJ7	2	3	5	2
YLJ8	2	2	4	0
YLJ9	3	3	4	1
YLJ10	1	1	3	0
YLJ11	2	5	6	4
YLJ12	2	2	3	1
YLJ13	2	2	4	0
YLJ14	2	4	5	1
YLJ15	2	3	5	2
YLJ16	2	3	4	1
YLJ17	2	2	4	1
YLJ18	3	3	5	1
**Total**	6	19	26	12

Table S3. Statistics of taxonomy annotation of zooplankton in Liaohe and Yalujiang estuaries.

Taxonomy	Phylum	Family	Genus	Species
LH1	6	10	9	4
LH2	5	6	6	3
LH3	3	5	7	6
LH4	1	3	4	2
LH5	1	4	7	4
LH6	1	6	5	3
LH7	4	7	6	4
LH8	3	6	7	4
LH9	4	6	6	3
LH10	2	4	3	2
LH11	2	6	6	4
LH12	6	7	5	3
LH13	3	4	3	1
LH14	2	4	4	2
LH15	2	5	4	2
YLJ1	3	6	6	3
YLJ2	3	4	5	2
YLJ3	3	5	7	3
YLJ4	3	6	8	5
YLJ5	2	4	6	4
YLJ6	4	6	9	6
YLJ7	4	6	10	5
YLJ8	4	6	10	5
YLJ9	3	4	4	1
YLJ10	4	6	9	6
YLJ11	5	8	8	3
YLJ12	4	5	6	2
YLJ13	3	6	7	4
YLJ14	3	5	8	5
YLJ15	2	4	7	4
YLJ16	3	4	5	2
YLJ17	2	4	6	4
YLJ18	4	6	9	7
**Total**	20	28	40	24

Table S4. Distance-decay similarities of phytoplankton and zooplankton for environmental similarity and geographic distance in Liaohe and Yalujiang estuaries.

**Community**	**Location**	**Factor**	**Slope**	**Intercept**	**R^2**	**p-value**
Phytoplankton	LH	ENV	0.260	0.279	0.004	0.233
		GEO	-0.006	0.619	0.048	**0.015***
	YLJ	ENV	0.002	0.864	-0.007	0.958
		GEO	-0.001	0.887	0.021	**0.042***
Zooplankton	LH	ENV	-0.015	0.315	-0.010	0.929
		GEO	-0.001	0.327	-0.007	0.573
	YLJ	ENV	0.247	0.228	0.025	**0.029***
		GEO	-0.005	0.512	0.082	**<0.001***

Table S5. Effects of environmental variables on variations in phytoplankton and zooplankton communities identified by canonical correlation analysis.

Environmental variables	Phytoplankton		Zooplankton	
	r^2	*p*-value	r^2	*p*-value
**Temperature**	0.361	**0.001**	0.563	**0.001**
**Salinity**	0.357	**0.003**	0.264	**0.013**
**DO**	0.152	0.104	0.225	**0.026**
**SS**	0.366	**0.006**	0.614	**0.001**
**COD**	0.804	**0.001**	0.887	**0.001**
**PHs**	0.690	**0.001**	0.747	**0.001**
**PO4**	0.274	**0.005**	0.423	**0.001**
**NO2**	0.141	0.113	0.010	0.218
**NO3**	0.204	**0.032**	0.342	**0.001**
**NH4**	0.641	**0.001**	0.488	**0.002**
**Cu**	0.564	**0.001**	0.531	**0.001**
**Pb**	0.500	**0.0018**	0.418	**0.004**
**Zn**	0.011	0.841	0.016	0.783
**Cd**	0.504	**0.003**	0.329	**0.028**
**Cr**	0.047	0.409	0.021	0.692
**As**	0.502	**0.001**	0.592	**0.001**

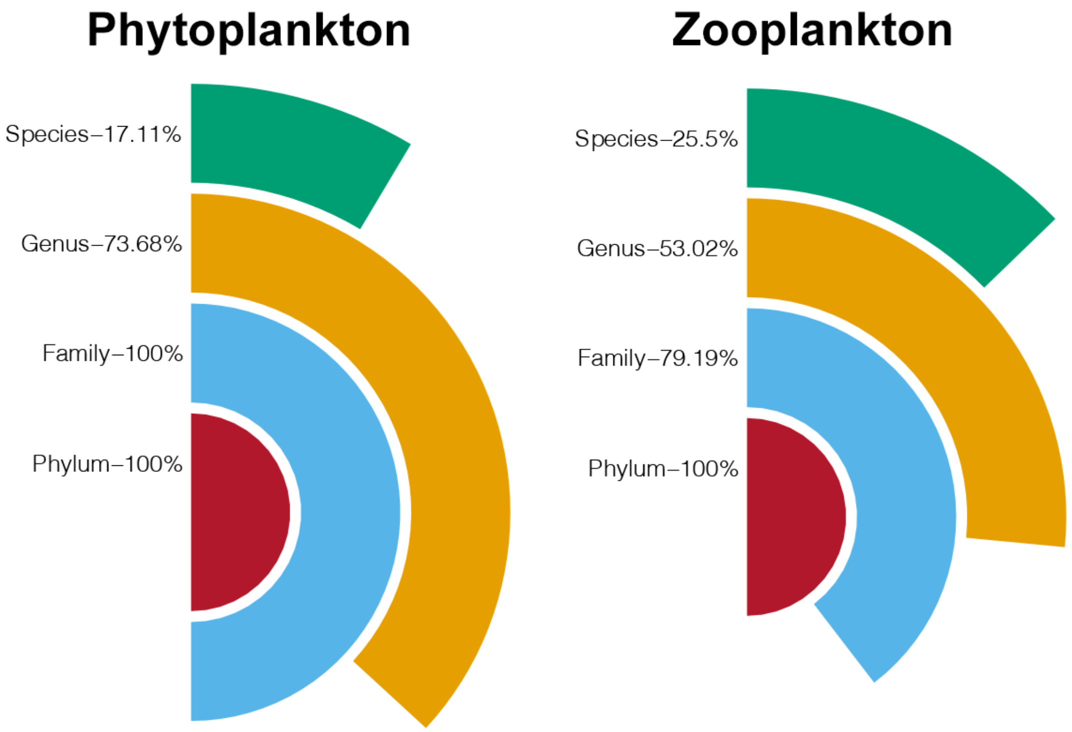

32

33 **Figure S1.** Ratios of successfully annotated ASVs at different taxonomic levels.

34

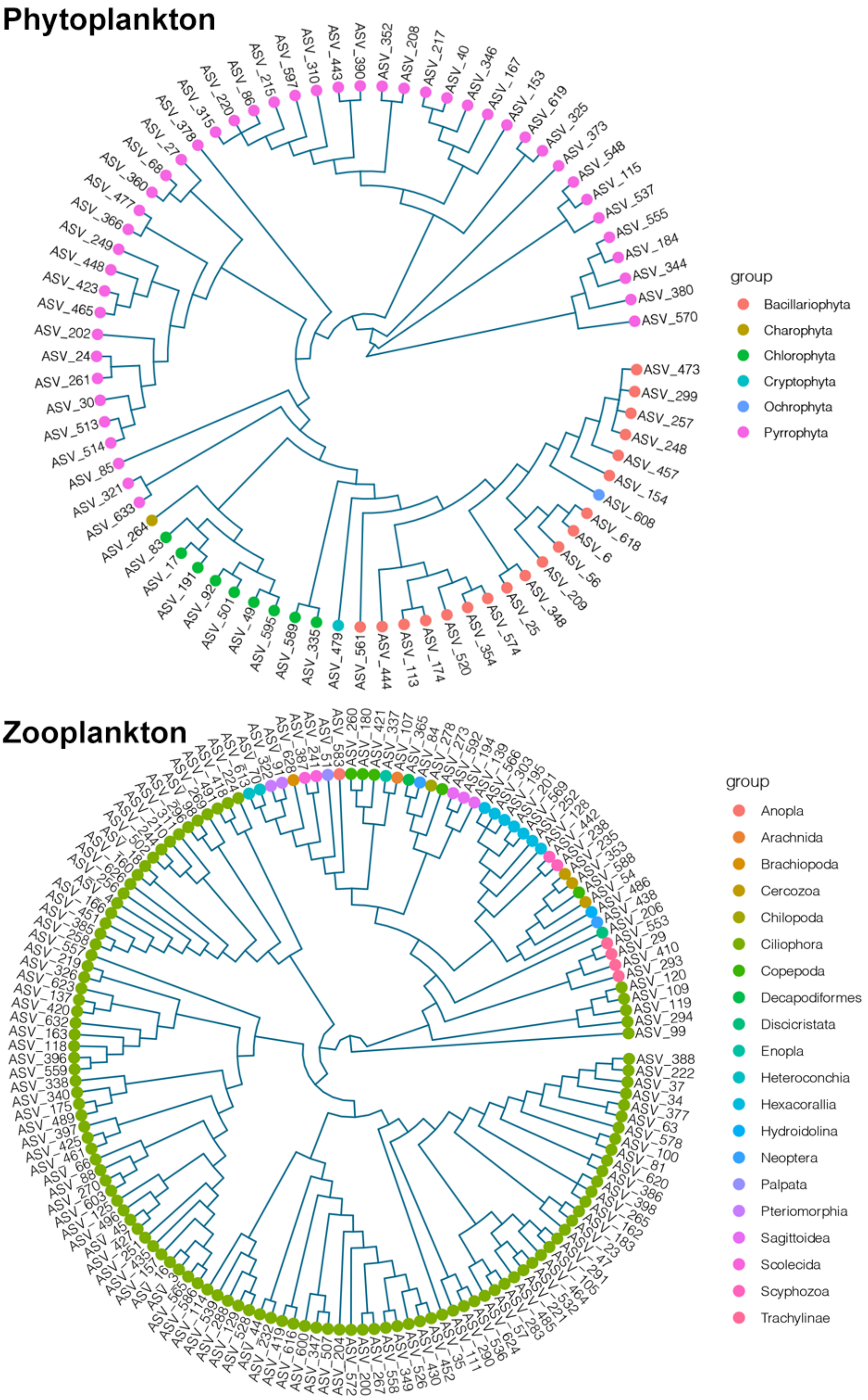

Figure S2. Phylogenetic trees of phytoplankton and zooplankton ASVs recognized in Liaohe and Yalujiang estuaries.

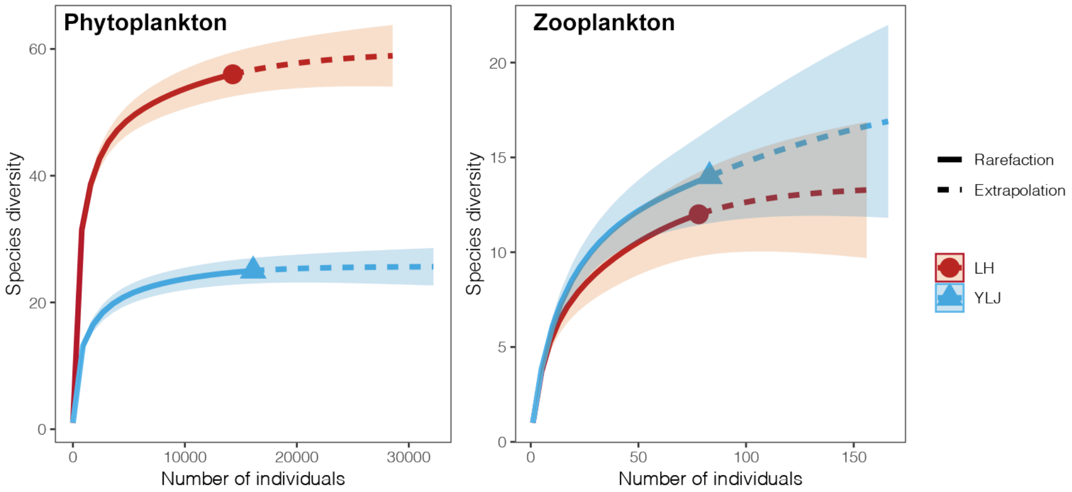

Figure S3. Rarefaction curves of phytoplankton and zooplankton in Liaohe (LH) and Yalujiang (YLJ) estuaries. The circular and triangular points represent the maximum number of points reached in the sampling.

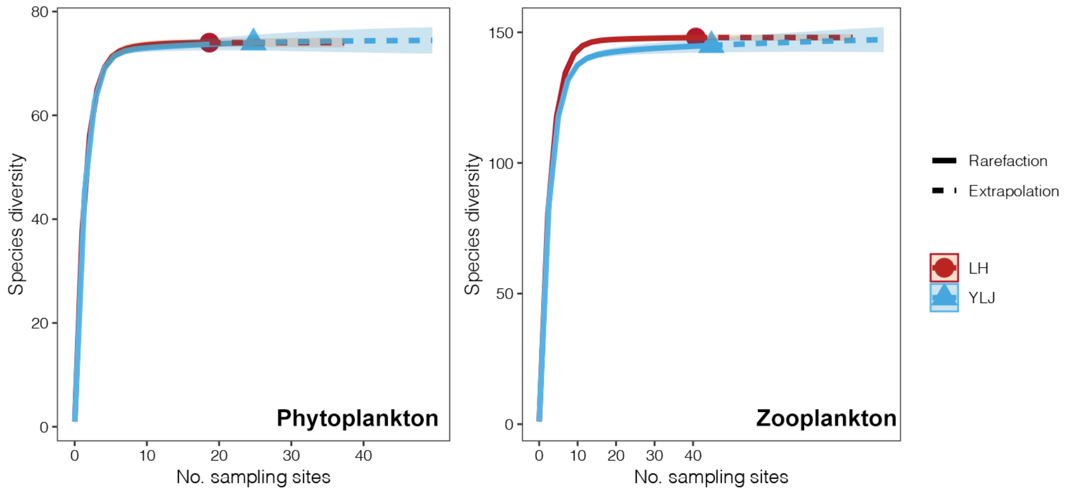

Figure S4. Cumulative curves of the number of phytoplankton and zooplankton ASV numbers detected in Liaohe (LH) and Yalujiang (YLJ) estuaries. The circular and triangular points represent the maximum number of points reached in the sampling.

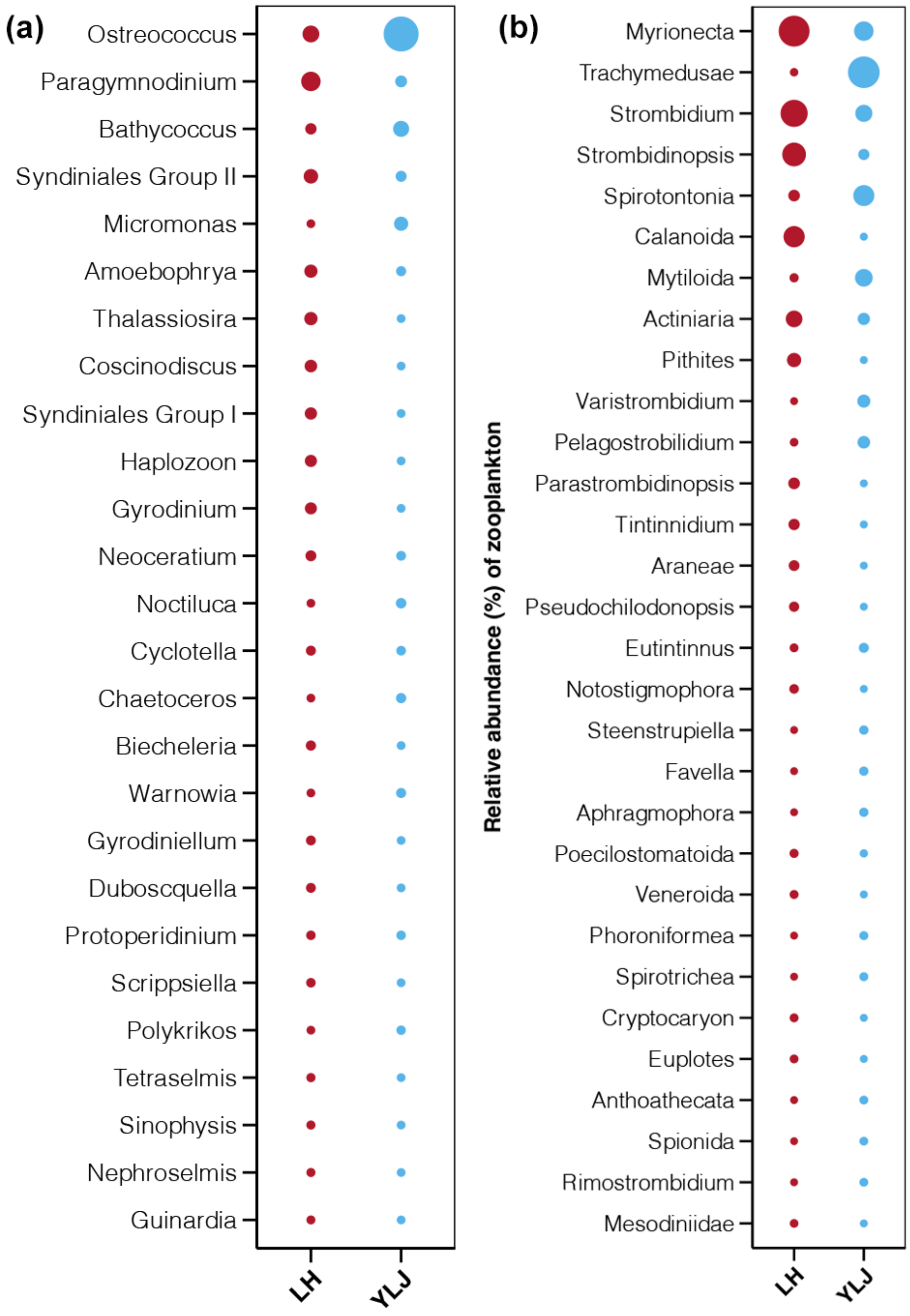

Figure S5. Relative abundances of top30 phytoplankton (a) and zooplankton (b) genera in Liaohe (LH) and Yalujiang (YLJ) estuaries. The size of the points represents the relative abundance of the genera.

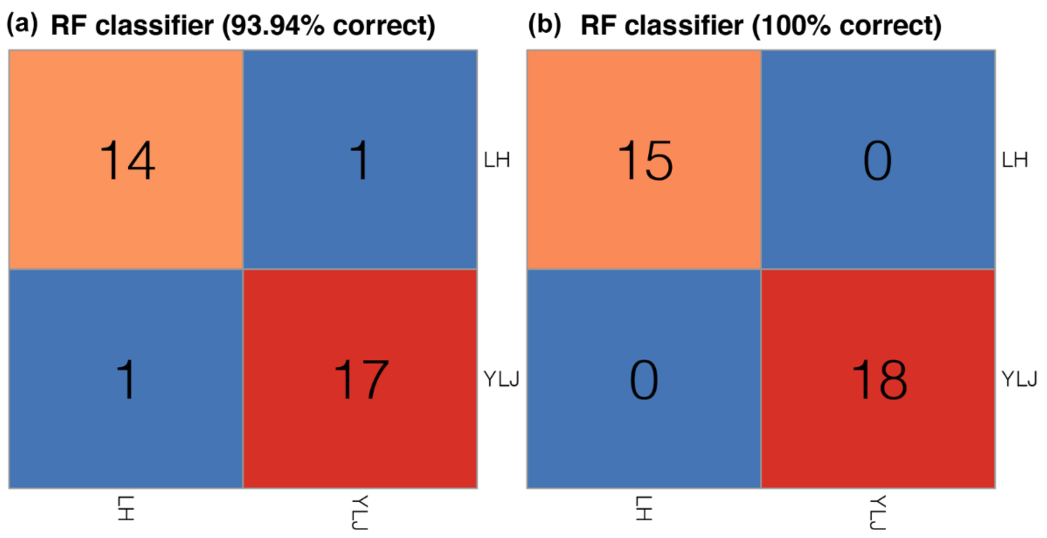

Figure S6. Accuracy of random forest models based on phytoplankton (a) and zooplankton (b) genera for distinguishing the origins of water samples. The numbers on the diagonal represent the number of samples in which the prediction results of random forest model are consistent with the actual results. The numbers in other cells represent the number of samples in which the name of the row is their actual region, but the random forest model predicts them as belonging to the region indicated by the name of the column.

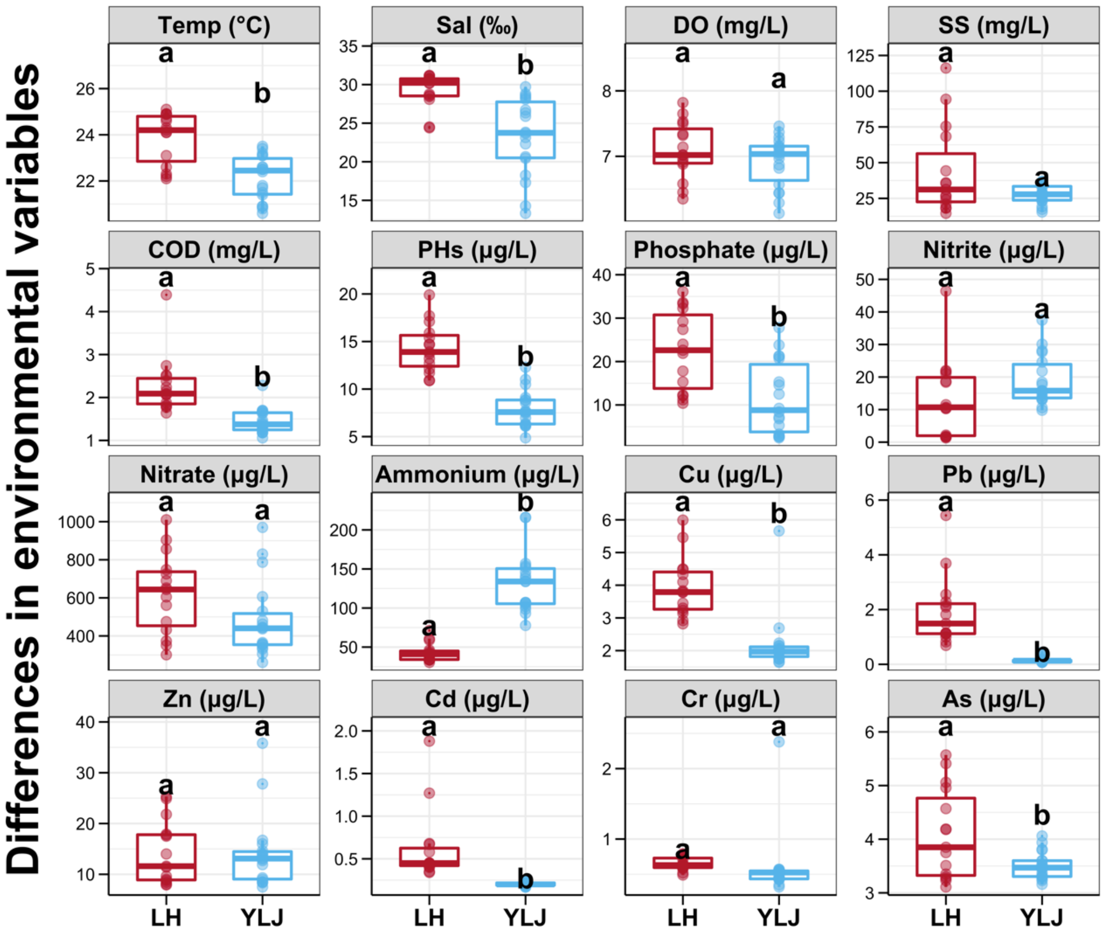

Figure S7. Differences in environmental variables between the LH and YLJ coastal areas. Different lowercase letters above each box in the same subfigure represent significant differences between groups (t-test, $p < 0.05$).

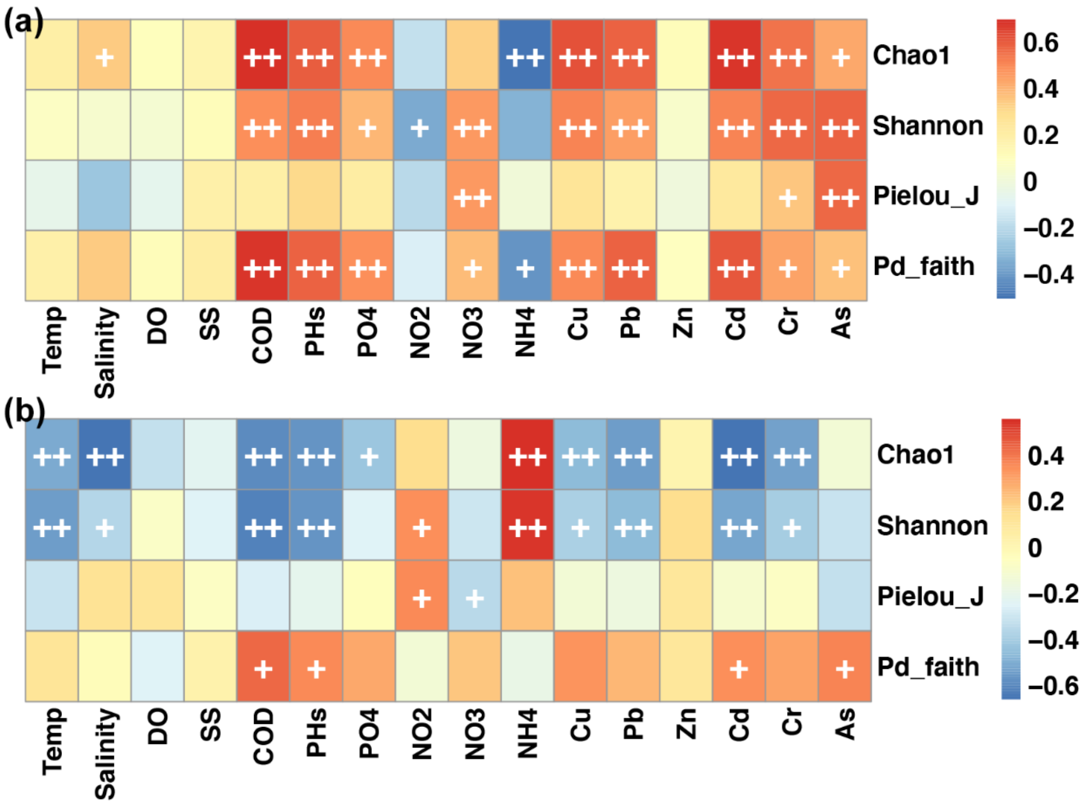

Figure S8. Heatmap of correlations between the alpha diversity indices of phytoplankton (a) and zooplankton (b) communities with environmental variables in the LH and YLJ estuaries. Spearman correlation coefficient was displayed by the color of each cell in heatmap. A significant correlation was confirmed if the p-value with Bonferroni adjustment was less than 0.05 (+) or 0.01 (++).

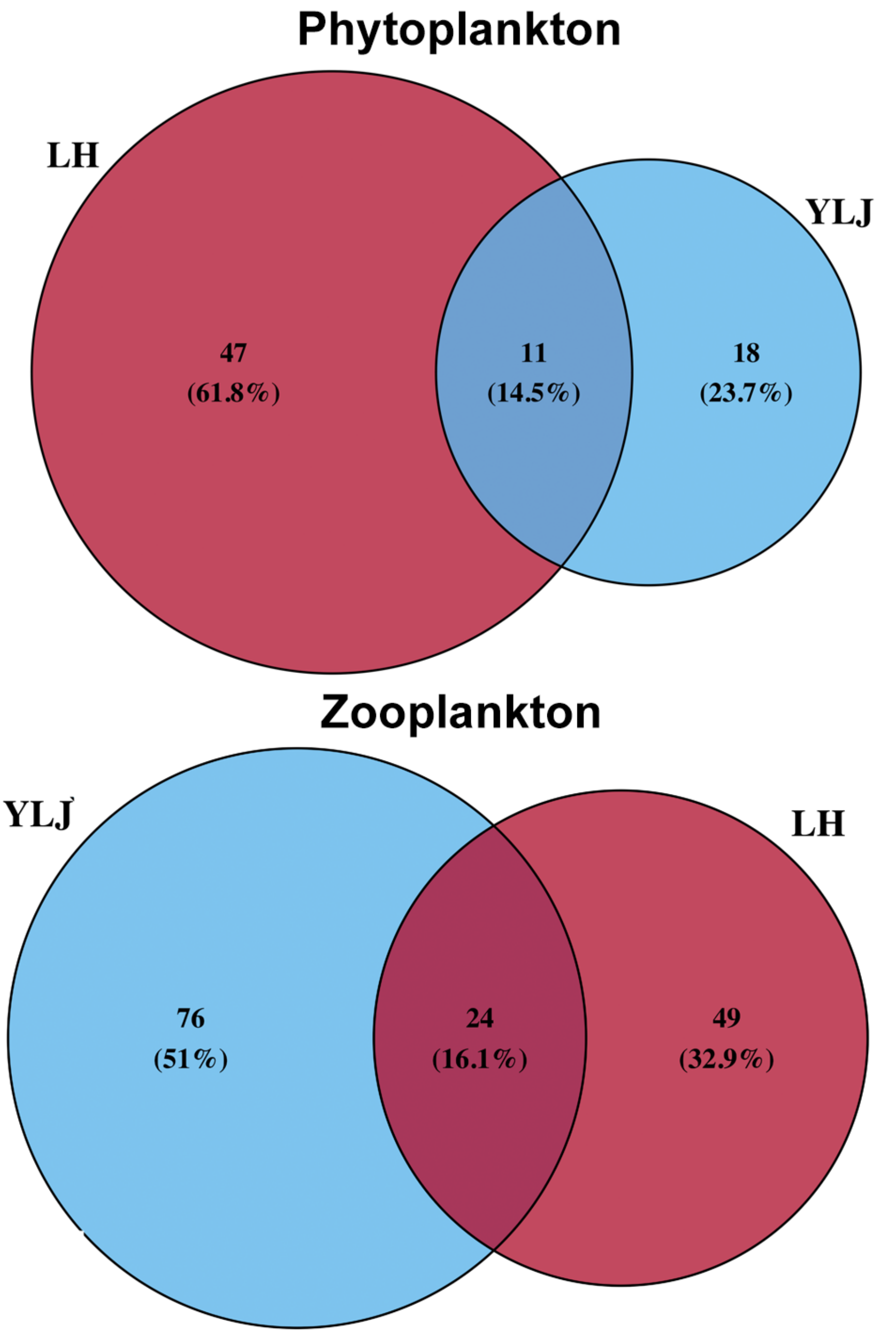

Figure S9. Shared and unique phytoplankton and zooplankton ASVs in LH and YLJ estuaries.
